# Visualizing the invisible: class excursions to ignite children’s enthusiasm for microbes

**DOI:** 10.1111/1751-7915.13576

**Published:** 2020-05-14

**Authors:** Terry J. McGenity, Amare Gessesse, John E. Hallsworth, Esther Garcia Cela, Carol Verheecke‐Vaessen, Fengping Wang, Max Chavarría, Max M. Haggblom, Søren Molin, Antoine Danchin, Eddy J. Smid, Cédric Lood, Charles S. Cockell, Corinne Whitby, Shuang‐Jiang Liu, Nancy P. Keller, Lisa Y. Stein, Seth R. Bordenstein, Rup Lal, Olga C. Nunes, Lone Gram, Brajesh K. Singh, Nicole S. Webster, Cindy Morris, Sharon Sivinski, Saskia Bindschedler, Pilar Junier, André Antunes, Bonnie K. Baxter, Paola Scavone, Kenneth Timmis

**Affiliations:** ^1^ School of Life Sciences University of Essex Colchester UK; ^2^ Department of Biological Sciences and Biotechnology Botswana International University of Science and Technology Palapye Botswana; ^3^ Institute for Global Food Security School of Biological Sciences Queen’s University Belfast Belfast UK; ^4^ School of Life and Medical Science University of Hertfordshire Hatfield UK; ^5^ Cranfield Soil and Agrifood Institute Cranfield University Cranfield UK; ^6^ School of Life Sciences and Biotechnology Shanghai Jiao Tong University Shanghai 200240 China; ^7^ Escuela de Química Centro de Investigaciones en Productos Naturales (CIPRONA) Universidad de Costa Rica San José Costa Rica; ^8^ Centro Nacional de Innovaciones Biotecnológicas (CENIBiot) CeNAT-CONARE San José Costa Rica; ^9^ Department of Biochemistry and Microbiology Rutgers University New Brunswick NJ USA; ^10^ Novo Nordisk Foundation Center for Biosustainability Technical University of Denmark Lyngby Denmark; ^11^ Institut Cochin 24 rue du Faubourg Saint‐Jacques 75014 Paris France; ^12^ Food Microbiology Wageningen University and Research Wageningen The Netherlands; ^13^ Department of Microbial and Molecular Systems Centre of Microbial and Plant Genetics Laboratory of Computational Systems Biology KU Leuven 3001 Leuven Belgium; ^14^ Department of Biosystems Laboratory of Gene Technology KU Leuven 3001 Leuven Belgium; ^15^ School of Physics and Astronomy University of Edinburgh Edinburgh UK; ^16^ Chinese Academy of Sciences Beijing 100101 China; ^17^ Department of Medical Microbiology and Immunology University of Wisconsin Madison WI USA; ^18^ Department of Biological Sciences University of Alberta Edmonton AB Canada; ^19^ Department of Biological Sciences Vanderbilt Microbiome Initiative Vanderbilt University Nashville TN USA; ^20^ The Energy and Resources Institute Lodhi Road New Delhi 110003 India; ^21^ Department of Chemical Engineering University of Porto 4200‐465 Porto Portugal; ^22^ Department of Biotechnology and Biomedicine Technical University of Denmark Lyngby Denmark; ^23^ Hawkesbury Institute for the Environment University of Western Sydney Penrith Australia; ^24^ Australian Institute of Marine Science Townsville QLD Australia; ^25^ Australian Centre for Ecogenomics University of Queensland Brisbane QLD Australia; ^26^ INRAE Pathologie Végétale 84140 Montfavet France; ^27^ Albuquerque NM USA; ^28^ Institute of Biology University of Neuchâtel Neuchâtel Switzerland; ^29^ State Key Laboratory of Lunar and Planetary Sciences Macau University of Science and Technology (MUST) Taipa, Macau SAR China; ^30^ Great Salt Lake Institute Westminster College Salt Lake City Utah USA; ^31^ Department of Microbiology Instituto de Investigaciones Biológicas Clemente Estable Montevideo Uruguay; ^32^ Institute of Microbiology Technical University of Braunschweig Braunschweig Germany

## Abstract

We have recently argued that, because microbes have pervasive – often vital – influences on our lives, and that therefore their roles must be taken into account in many of the decisions we face, society must become microbiology‐literate, through the introduction of relevant microbiology topics in school curricula (Timmis *et al.* 2019. *Environ Microbiol* **21:** 1513‐1528). The current coronavirus pandemic is a stark example of why microbiology literacy is such a crucial enabler of informed policy decisions, particularly those involving preparedness of public‐health systems for disease outbreaks and pandemics. However, a significant barrier to attaining widespread appreciation of microbial contributions to our well‐being and that of the planet is the fact that microbes are seldom visible: most people are only peripherally aware of them, except when they fall ill with an infection. And it is disease, rather than all of the positive activities mediated by microbes, that colours public perception of ‘germs’ and endows them with their poor image. It is imperative to render microbes visible, to give them life and form for children (and adults), and to counter prevalent misconceptions, through exposure to imagination‐capturing images of microbes and examples of their beneficial outputs, accompanied by a balanced narrative. This will engender automatic mental associations between everyday information inputs, as well as visual, olfactory and tactile experiences, on the one hand, and the responsible microbes/microbial communities, on the other hand. Such associations, in turn, will promote awareness of microbes and of the many positive and vital consequences of their actions, and facilitate and encourage incorporation of such consequences into relevant decision‐making processes. While teaching microbiology topics in primary and secondary school is key to this objective, a strategic programme to expose children directly and personally to natural and managed microbial processes, and the results of their actions, through carefully planned class excursions to local venues, can be instrumental in bringing microbes to life for children and, collaterally, their families. In order to encourage the embedding of microbiology‐centric class excursions in current curricula, we suggest and illustrate here some possibilities relating to the topics of food (a favourite pre‐occupation of most children), agriculture (together with horticulture and aquaculture), health and medicine, the environment and biotechnology. And, although not all of the microbially relevant infrastructure will be within reach of schools, there is usually access to a market, local food store, wastewater treatment plant, farm, surface water body, etc., all of which can provide opportunities to explore microbiology in action. If children sometimes consider the present to be mundane, even boring, they are usually excited with both the past and the future so, where possible, visits to local museums (the past) and research institutions advancing knowledge frontiers (the future) are strongly recommended, as is a tapping into the natural enthusiasm of local researchers to leverage the educational value of excursions and virtual excursions. Children are also fascinated by the unknown, so, paradoxically, the *invisibility* of microbes makes them especially fascinating objects for visualization and exploration. In outlining some of the options for microbiology excursions, providing suggestions for discussion topics and considering their educational value, we strive to extend the vistas of current class excursions and to: (i) inspire teachers and school managers to incorporate more microbiology excursions into curricula; (ii) encourage microbiologists to support school excursions and generally get involved in bringing microbes to life for children; (iii) urge leaders of organizations (biopharma, food industries, universities, etc.) to give school outreach activities a more prominent place in their mission portfolios, and (iv) convey to policymakers the benefits of providing schools with funds, materials and flexibility for educational endeavours beyond the classroom.

## Graphical abstract

The ubiquity of microbes, their manifold activities and pervasive influence on the health of all life, local environments and the planet, necessitate an understanding of relevant microbial processes for informed, evidence‐based decision‐making at all levels of society – i.e. Microbiology Literacy. While teaching microbiology topics in school is key to this objective, a strategic program to expose children directly and personally to natural and managed microbial processes, and the results of their actions, through carefully planned class excursions to local venues, can be instrumental in bringing microbes to life for children. In outlining some of the options for microbiology excursions, providing suggestions for discussion topics, and considering their educational value, we strive to extend the vistas of current class excursions and to: (i) inspire teachers and school managers to incorporate more microbiology excursions into curricula; (ii) encourage microbiologists to support school excursions and generally get involved in bringing microbes to life for children; (iii) urge leaders of organisations (biopharma, food industries, universities, etc.) to give school outreach activities a more prominent place in their mission portfolios, and (iv) convey to policy makers the benefits of providing schools with funds, materials and flexibility for educational endeavours beyond the classroom.

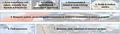

## Introduction

The ubiquity of microbes, their manifold activities and pervasive influence on the health of all life, local environments and the planet, necessitate an understanding of relevant microbial processes for informed, evidence‐based decision‐making at all levels of society. These range from the individual (e.g. relating to diet, hygiene, vaccination, pet ownership, choice of domestic cleaning products) through to governments and international organizations (e.g. provision of clean water, managing climate change, food security, monitoring and controlling the spread of pathogens and antibiotic resistance) (Timmis *et al.*, 2019). Microbial products and activities are pivotal to remedying major societal problems (e.g. by degrading toxic pollutants and waste, attaining increased crop yields, producing biodegradable plastics, ensuring the supply of new medicines, including antibiotics, and providing sustainable fuels), and so are key to policy decisions aimed at achieving Sustainable Development Goals (see editorial by Timmis *et al.*, 2017a, and papers in the associated Special Issue of *Microbial Biotechnology*), including the provision of opportunities for entrepreneurship, economic growth and employment generation (Timmis *et al.*, 2017b). Microbes provide life‐supporting services to the humans, other animals and plants that they inhabit, and constitute Earth’s life‐support system by driving biogeochemical cycles. Consequently, microbiology is an exceptionally dynamic research field, with major discoveries being made at frantic speed. New understanding thereby acquired constitutes an increasingly reliable foundation that can aid decision‐making at all levels, but, in order to connect understanding and decision‐making, the attainment of microbiology literacy in society is needed.

At the heart of the advancement of microbiology literacy is the education and inspiration of children, who typically have a voracious appetite for knowledge and are readily stimulated by the dynamism and excitement of the topic. Realization of the existence of their own microbiomes, and the intimacy of the relationships children have with their microbial friends, will captivate them, stimulate their natural curiosity and fire their imagination and passion.

However, there exists a major impediment to teaching children about microbes, namely their microscopic size, which prevents them being portrayed by the teacher and noticed in the environment when children are out and about (exceptions include fungal fruiting bodies, lichens, eutrophic waters and biofilms). Microbes are thus essentially abstract entities that are not apparent as being majority partners of the communities in which we live, and responsible for the many processes occurring in and around us. It is paramount that we enable children to conjure up mental images of microbes, in order to establish and appreciate the links between natural and human‐managed microbial processes, on the one hand, and the underlying microbes and their activities, on the other hand.

**Fig. 1:**
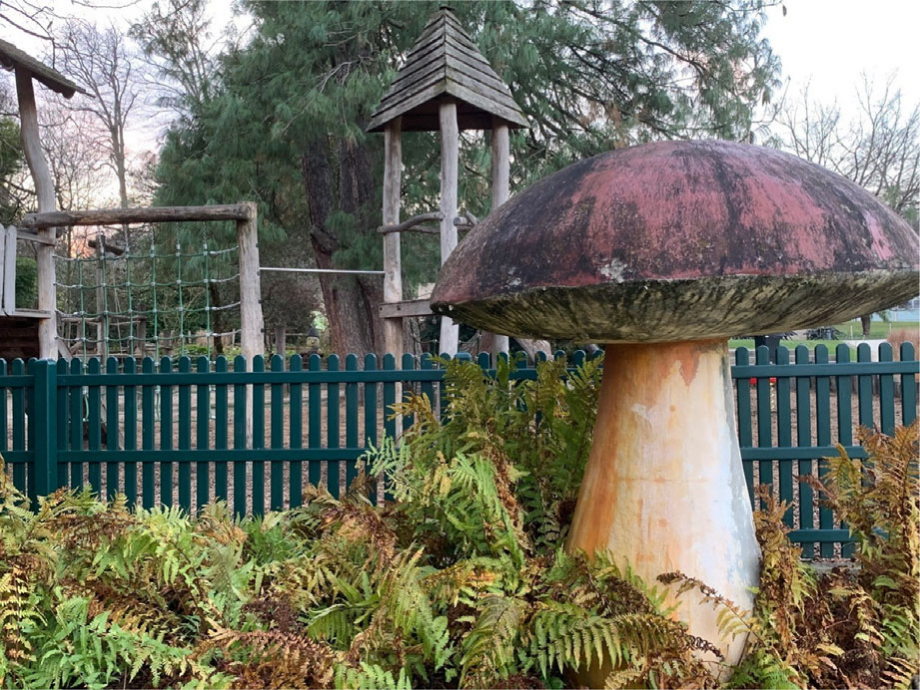
Mushrooms go hand in hand with folklore creatures in the playground of the Geneva Botanical Garden. *Photograph by Kenneth Timmis*

Young children’s emotional attachment to ‘cute’ animals and teddy bears is predicated on their being visible and in most cases touchable/cuddlable. Of course, mushrooms, as macroscopic manifestations of microbial subunits, are generally more recognizable, with mushroom‐mimicking decorative objects and furniture being ubiquitous in children’s play areas (Fig. 1), but this is the exception.

And, as we have stated (Timmis *et al.*, 2019), it is essential for microbes to ‘*transit from abstraction to pictorial perception and substance, and take up their rightful position in the human psyche. Visual aids will thus take centre stage …. and the exploding arena of microbial art will stimulate the imagination….As microbes transit from the abstract and take form, they will become real; children will have their favourites! Cuddly teddy bears and woolly sheep will be joined by steamy Methano, wily Wolbo and prickly Diatoma, who all have their individual (anthropocentric) characters assigned by agile toy manufacturers*’. Real but invisible microbes become visible, cuddlable toys with names. They may even become characters in storybooks and TV cartoons, with goodies and baddies drawn from the real microbial world.

While toys and artwork will bring microbes to life and give them form for younger children, and in some cases inform about individual microbial functions, natural and managed microbial activities will remain essentially abstract. In order to remedy this deficit, it is crucial to expose children to tangible, readily comprehended examples of important microbial activities. Time‐honoured mechanisms for this are class experiments and organized class outings/excursions, which allow children to personally experience and investigate objects and processes in their natural settings. Class excursions are a particularly enjoyable means of creating links between microbes and their activities, of making microbial activities tangible and of immersing children in microbiology.

By introducing children to workplaces, operational plants or field sites, excursions can demonstrate the integrated nature of topics and bring reality to learning, thereby stimulating natural curiosity (see Knapp, 1996; Behrendt and Franklin, 2014), which may be especially pronounced in those who do not thrive in the traditional classroom (Ofsted, 2008). Microbiology excursions may reap extra benefits when taking place in natural surroundings, for which there is growing evidence of improved learning, as well as encouragement to develop a sense of responsibility for environmental stewardship (Ballantyne and Packer, 2002; Kuo *et al.*, 2019). Introducing children to microbiology beyond the classroom via organized events also provides a bridge to, and enriches, their informal learning from everyday environments and activities (Bell *et al.*, 2009), such as watching television (e.g. images of the sea: remembering microbes make up 65% of marine biomass; Bar‐On *et al.*, 2018), going for a walk (e.g. noticing the diversity of the lichens on walls and roofs) and visiting the shops (e.g. *yeast made that baguette!*) or a museum (e.g. the dark patches on that old document might be fungal growth).

Class excursions are not only educational and fun events for children and teachers alike, but also invaluable introductions to future professional options, which can lead to career orientation, summer jobs, internships and contacts. They also provide a different educational environment and expose pupils to new “teachers” with a diversity of personalities and teaching styles, which can be enriching.

**Fig. 2:**
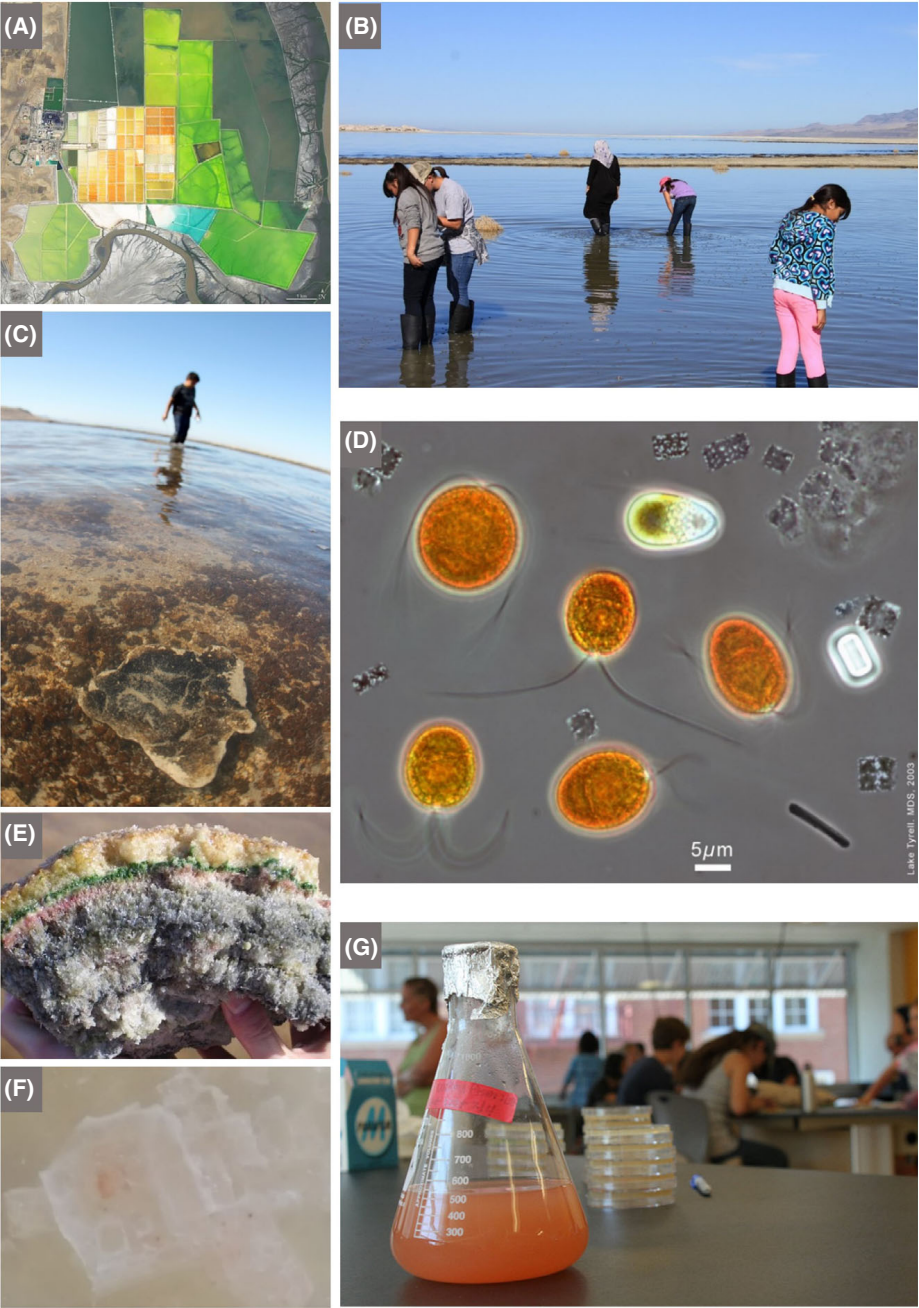
Exploring hypersaline environments and extremely halophilic microbes. Their vivid colours, extreme adaptations, biotechnological applications and ease of growth make halophiles very attractive to children. The manufacture of sea salt, with its pivotal role in food preservation, helped regions and civilizations to flourish. Thus, the interconnectedness of microbes, food and history can be explored by visiting hypersaline environments or when buying sea salt and salted foods from local shops A. Satellite view of the salterns in Bhavnagar, India, showing vivid red, orange and green ponds in which seawater evaporates leaving behind salt (field of view is ~ 10 km^2^). The colours are due to the pigments of the halophilic microbes in the brines of different salinity. The red/orange pigments of haloarchaea and *Dunaliella salina* (see D) encourage the absorption of solar radiation leading to increased rates of evaporation, resulting in more rapid salt production. B. A teacher and her class wade into Great Salt Lake (Utah, USA) to collect samples to study under field microscopes. C. A student investigates the biofilms that form stromatolite‐like structures in Great Salt Lake, impressive calcium carbonate deposits precipitated by the actions of cyanobacteria. D. Microscopic image from the hypersaline Lake Tyrrell, Australia (salinity> 20% w/v), in which we can tentatively identify the eukaryotic chlorophyte, *Dunaliella salina* (grown commercially for the carotenoid, β‐carotene, which is widely used as a natural food colorant as well as a precursor to vitamin A)*,* living alongside the haloarchaeon, *Haloquadratum walsbyi,* which has flat square‐shaped cells with gas vesicles that allow flotation to the surface, most likely to acquire oxygen (scale bar is 5 μm). E. Gypsum crust from the bottom of a shallow saltern pond (~20% w/v salinity) in Eilat, Israel, showing layered microbial communities of phototrophic microbes. The orange‐brown upper layer is most likely dominated by unicellular cyanobacteria; the green layer by filamentous cyanobacteria; and the purple layer by anoxygenic purple sulfur bacteria. These microbes use light as a source of energy, and their layering is explained by differential tolerance to ultraviolet light and oxygen, and their capacity to use light of different wavelengths in photosynthesis. The anoxygenic purple sulfur bacteria are adapted to use light at the far‐red end of the spectrum, which is less attenuated than visible light in sediments, and they benefit by living in close proximity to anaerobic sulfate‐reducing bacteria (grey layer directly beneath the purple layer), which produce hydrogen sulfide. This compound is split to form oxidized sulfur in a similar way to which the oxygenic phototrophs, like cyanobacteria, produce oxygen from water. Such layering of microbial communities can trigger discussions about chemistry (the electron donors and products), physics (diffusion of oxygen and hydrogen sulfide, and the electromagnetic spectrum), geology (microbial fossils in gypsum) and ecological concepts (interspecies interactions and niche partitioning). F. A laboratory‐made salt (halite) crystal coloured red due to the presence of haloarchaea trapped inside small pockets of brine within the crystal (the central crystal is about 0.5 by 0.5 cm). This is a survival strategy used by haloarchaea to avoid desiccation. They remain viable inside the halite, and evidence suggests that some haloarchaea can survive over geological time inside buried halite. Some haloarchaea play a major role in hydrolysing biopolymers in salty environments, such as those used in the production of fish sauces. When we consume the sauces or sea salt, we consume haloarchaea! G. During an excursion, students can collect samples from which they can inoculate media (in this case to grow extreme halophiles) after returning to the classroom, bringing the field‐collected microorganisms into the laboratory and further connecting students to their environment. *Photograph A by NASA* https://earthobservatory.nasa.gov/ *. Photographs B, C and G by Great Salt Lake Institute. Photograph D by Mike Dyall‐Smith. Photograph E by Andreas Thywißen.*

Bringing microbes to life through class excursions can strongly stimulate children’s imagination and emotions by teachers exposing some of the more exceptional or extraordinary features of microbes and their manifestations, such as: being the first forms of life to evolve on, inhabit and populate, the Earth (and likely the last); their ability to grow in extreme environments (hot springs, ice, deep subsurface and inside salt crystals; Fig. 2) and the question of whether they may exist on other planets (and, indeed, whether life on Earth could have been ‘seeded’ from such microbe‐populated planets – panspermia?); the ubiquity and tenacity of microbes and the fact that they follow us into space, on our bodies and space craft; microbes contributing to about half the oxygen we breathe, driving evolution of ‘higher organisms’ – the Eukaryotes – and thus humans (e.g. via mitochondria and chloroplasts); their presence in and on humans in vast numbers, roughly equal to the number of human cells (and influencing our well‐being); inherent beauty (e.g. *Thiomargarita namibiensis,* one of the largest known Bacteria; Schulz 2002); unusual adaptations (e.g. *Magnetospirillum magneticum* that contains nanomagnets to navigate using Earth's geomagnetic field; Chen *et al.*, 2010); and other enigmatic forms (e.g. flat, square archaeal species, *Haloquadratum walsbyi;* Fig. 2).

With this Editorial, we strive to promote class excursions in practical microbiology and, to both facilitate this end and encourage teachers to consider available options, provide a non‐exhaustive list of possible excursions and microbial processes or products to explore. Local knowledge, along with an understanding of the role of microbiology in the processes listed below, is obviously key to identifying the most stimulating school trips and activities. We also provide some suggestions on the preparation of excursions, to maximize enjoyment and information acquisition by the class, and optimize class–venue interactions.

During the preparation of this Editorial, SARS‐CoV‐2 started to make its own global excursion into and through the human population with devastating consequences, causing many thousands of deaths in the process. Obviously, social distancing measures put in place to slow the spread of the virus make school excursions at the time of writing undesirable and, in most countries, impossible (perhaps making this the most untimely of editorials!). Nevertheless, the pandemic will pass, leaving children with many questions about how pathogens are transmitted across species and from person to person, how transmission can be reduced or interrupted, why infected people are affected to different extents, and how disease can be prevented or treated. It is certain that the COVID‐19 pandemic will have a major impact on children, from many different angles, and create a generation of youngsters who are much more aware of, and curious about, microbiology. It is therefore more important than ever to harness this curiosity by providing opportunities to get a balanced view of the microbial world.

## Suggestions for class excursions

**Fig. 3:**
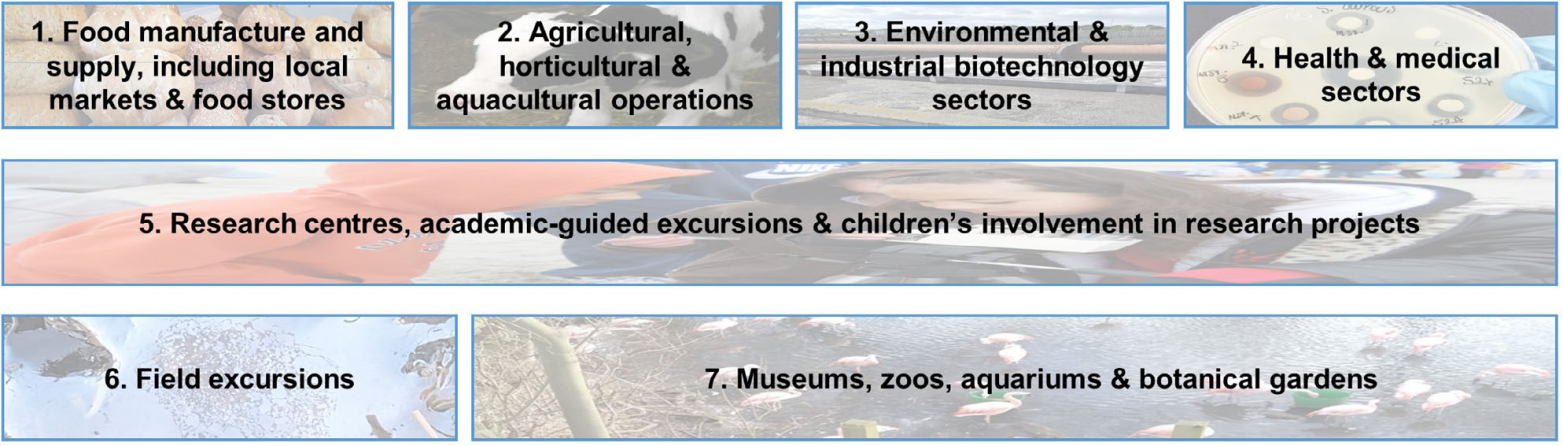
Excursion categories discussed in the text

The suggestions given below for class excursions are sorted for convenience into seven categories (Fig. 3), but many activities and associated discussion topics will be applicable across multiple headings. Moreover, identifying associations between categories should be encouraged.

### Food manufacture and supply, including local markets and food stores

#### Context

An adequate supply of good‐quality food is essential for human health, and ‘End hunger, achieve food security’ is a key UN Sustainable Development Goal (Willett *et al.*, 2019). Furthermore, food and drink contribute to human culture, civilization and *joie de vivre*. And, not entirely irrelevant for this discourse, food is a topic of passionate interest for most children. For many foods, microbes play a pivotal role in their manufacture, enhancement of nutritional value, preservation, and potential spoilage or transmission of food‐borne disease. And, in turn, our diet greatly influences our microbiome, with consequences for our health (Kolodziejczyk *et al.*, 2019). Thus, there is the critical *food–microbes–health* nexus to explore and the personal decisions that result from it. Then, there is a growing awareness of the link between food choice and global crises like climate change, the *livestock–greenhouse gas production–global warming* nexus, leading to decisions about changes in behaviour, such as reducing meat consumption. Children are of course engaged, sometimes passionately, in the global warming debate, and some are leading efforts for policy changes. Many of them are aware of the *food–climate* connection and so are motivated to learn about food, the contributions of microbes in the food industry, interactions between food and the gut microbiome and their ecophysiological health consequences.

Fermented foods were probably the first processed foods. They proved to be a key element of human development and expansion, owing to their capacity to preserve cereal grains, legumes, vegetables, milk and meat, often improving nutritional value and providing food year‐round. Out of necessity, and no doubt an element of chance, grew a plethora of fermented foods and drinks, with a diversity of fascinating flavours, textures, colours and smells that became part of the cultural heritage of a society or region. Cheese is a particularly instructive example, with its enormous range of variations and combining historical (cheese manufacture is thought to have originated at least 7500 years ago), cultural, culinary (there are many ways of using cheese in food preparation) and olfactory aspects. Fermented foods can readily be used in school teaching, to understand not only the underlying microbiology, but also their importance in diet and human culture, and the key issues of food preservation and safety.

#### Suggestions

**Fig. 4:**
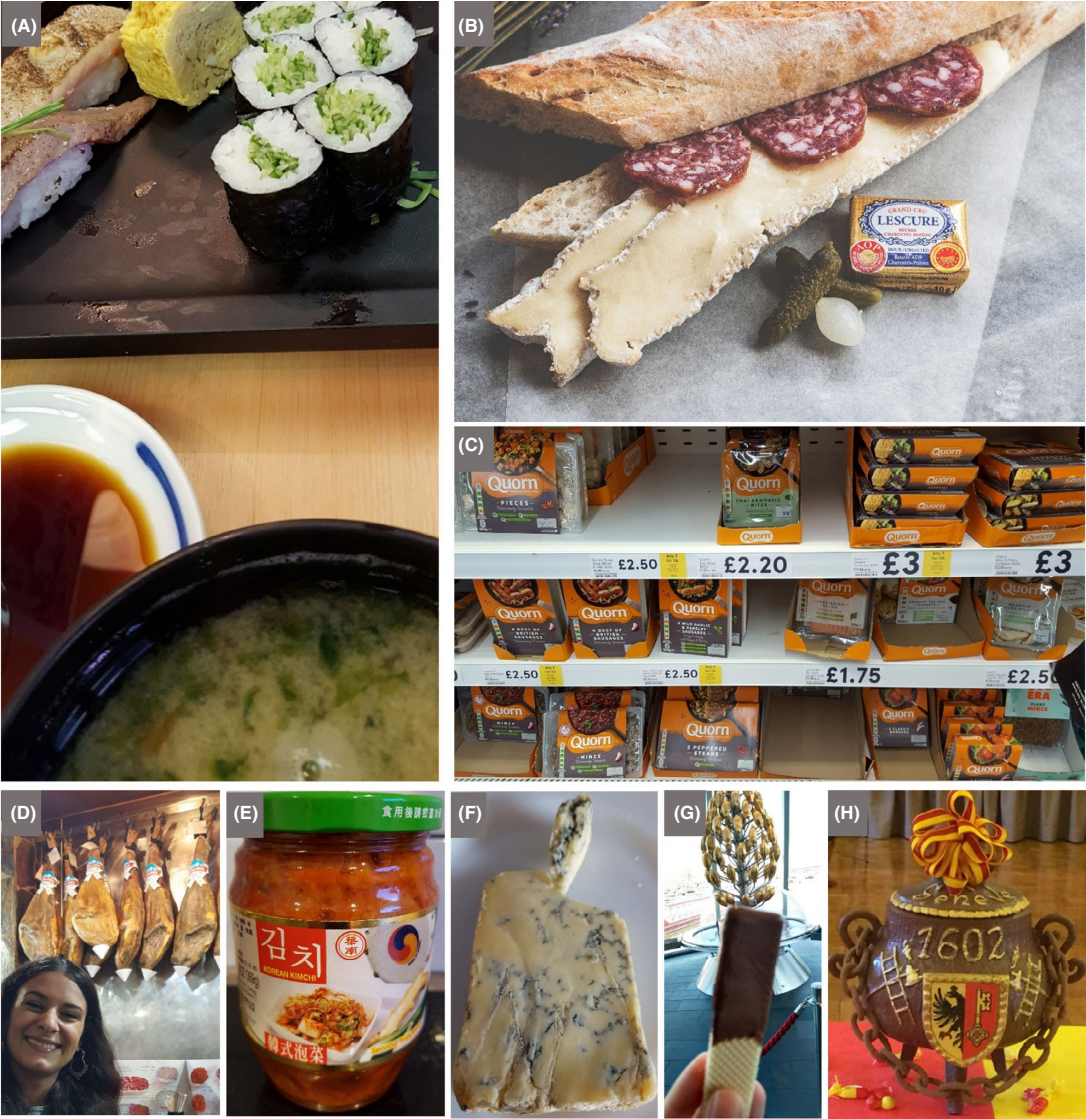
A trip to the food store or a meal can become an adventure of microbiological discovery A. Breakfast at Tsukiji Market, Japan. The miso in miso soup is a traditional Japanese paste produced by fermenting salted soya beans with kōji, which is also made by fermentation of rice or barley with the fungus *Aspergillus oryzae*. Soy sauce is made by fermenting salted soya beans and wheat by a complex community of hydrolytic fungi and bacteria, as well as lactic acid bacteria. Sushi rice is prepared with a sweet rice wine vinegar (mirin) to give it a delicate, sweet but sharp flavour. Vinegar can be prepared from almost any sugary solution by an alcoholic fermentation followed by an acetic fermentation. In Japan, vinegar traditionally derives from rice and kōji‐fermented rice. B. Salami and Brie baguette with pickles. Nearly all the foods in this image require microbes for their production: Bakers’ yeast (*Saccharomyces cerevisiae*) makes the dough rise for the bread; *Penicillium* species and lactic acid bacteria are among the microbes involved in both salami and Brie production; acetic acid‐producing bacteria make the vinegar from alcoholic drinks for the pickles. C. The drive towards reducing meat in the diet has opened a market for a range of meat‐free products, leading to a resurgence in the sales of *Quorn*, which is made from filamentous mycelium of the soil fungus *Fusarium venenatum*. D. Dry‐cured hams in a bar in Salamanca, Spain. Spanish ham is traditionally cured in brine with sea salt, often with added sodium nitrate, to lower the water activity to an extent that prevents the growth of spoilage organisms. E. Kimchi, a staple food in Korea, is becoming popular worldwide. Cabbage, along with numerous variations of vegetables and spices, is salted and undergoes fermentation dominated by lactic acid bacteria. The health benefits of this tangy, salty and often spicy product are widely reported (Tamang *et al.*, 2016). F. Stilton cheese, which, in addition to requiring lactic acid bacteria and other microbes in its manufacture, is inoculated with *Penicillium roqueforti* to produce the blue veins that contribute to the distinctive flavour. G. Chocolate from the Lindt factory in Cologne, Germany – an enjoyable way to learn about the history and microbiology of chocolate. H. Chocolate is moulded into a multitude of artistic forms that titillate the senses, especially of children. The example shown is that of the Geneva ‘marmite’, a chocolate representation of a soup cauldron containing marzipan vegetables, made each year for the festival of ‘Escalade’, the celebration of the defeat of an assault on the city in 1602. Legend has it that an old lady (Catherine Cheynel or *Mère Royaume*), who was making vegetable soup at night, spotted the attackers who were scaling the city walls, sounded the alarm and threw the hot soup cauldron at them. Incidentally, *Marmite* – the yeast extract spread putatively developed by a German scientist, Justus von Liebig, using leftover yeast from the brewing of beer in Burton upon Trent, UK – derives from the French name ‘marmite’, as it is sold in pots with a similar shape to the cauldron. *Photographs A and C‐F by Terry McGenity, B by Jez Timms on Unsplash, G by Mairi McGenity and H by Kenneth Timmis.*

Visits to local markets, food stores and food companies will allow children to explore a wide range of products (in addition to fermented foods) and ingredients that derive from microbes or their processes, including microbes as food (fungal *Quorn*, cyanobacterial *Spirulina*), diverse microbial metabolites (Kallscheuer, 2018) that are added to food (amino acids, vitamins, extracellular polysaccharides, acetic acid and citric acid) and enzymes (Raveendran *et al.*, 2018) (Fig. 4). In addition to the organizations listed below, universities and research centres with specialists in food microbiology (see Section 5) could be visited or contacted to request advice about food manufacturers in the local area.

- Manufacturers of fermented food:
  ◦ Dairy products, such as yoghurt, cheese, kumis and kefir
  ◦ Meat products, such as salami, nem chua and kargyong
  ◦ (Shell)fish products, such as fish sauces (nam pla, jeotgal, nuoc cham, ngari and Worcestershire sauce) and fish (surströmming and hongeo‐hoe)
  ◦ Vegetables, such as kimchi, sauerkraut and olives
  ◦ Roots, such as cassava products (mocaf and fufu)
  ◦ Legumes, such as soya bean pastes/sauces (tempeh, miso, doenjang, natto, kinema and soy sauce), pastes from the African locust bean tree (soumbala and iru) and others (lupin tempeh)
  ◦ Cereals, such as dosa, tape, ang‐kak and ogi
  ◦ Speciality food and drink, such as chocolate, coffee and kombucha
  ◦ Alcoholic beverages, such as beer (from large‐scale barley‐based brews to local millet‐ or agave‐based brews), sake, wine and distilled drinks
- Bread manufacturers
- Vinegar manufacturers
- Vegetarian and health food manufacturers, such as starter cultures for fermented food and drink, probiotics and prebiotics, algae, cyanobacteria, *Quorn* and yeast derivatives (e.g. *Marmite, Vegemite and Cenovis*)
- Manufacturers of food supplements, such as vitamins and amino acids (and artificial sweeteners derived from amino acids)
- Manufacturers of food and drink additives, such as flavours and flavour enhancers (e.g. glutamic acid and vanillin), colours (e.g. carotenoids and riboflavin), antioxidants (e.g. citric acid) and thickeners (e.g. xanthan gum and glycerol)
- Manufacturers of high‐fructose corn syrup
- Food microbiology quality control laboratories (e.g. of food manufacturers, abattoirs, food processors, food importers and food safety agencies)
- Food standard agencies (e.g. pathogen detection, detecting food crime and food provenance)

#### Some discussion topics

During a visit to a food store, children can be challenged to identify the microbial contribution to the food’s production process and its ingredients (Fig. 4). For example, peering at the fresh/frozen pizza offering provides considerable opportunity to explore microbial involvement in the production of this favourite food (yeast in the rising of the dough base, lactic acid bacteria in cheese production and salami ripening, sliced mushrooms and microbial fermentation of olives). The issue of sourcing food more locally and rejuvenation of the traditional preservation methods of fermentation, which add nutritional value and limit food waste (Campbell‐Platt, 1994; Brüssow, 2007; Katz, 2012; Tamang *et al.*, 2016; El Sheikha, 2018; Willett *et al.*, 2019), are fascinating topics to explore with children. Specifically, they can be asked to identify various means, in addition to fermentation, of reducing microbial spoilage (i.e. pickling with vinegar, adding sugar or salt, drying, freezing, refrigerating, vacuum packing and canning) and to discuss how they work. This exercise can be followed up at school with relatively simple experiments to test student‐led hypotheses about the methods that best preserve the food while maintaining its nutritional value. A wonderful way to learn about microbial fermentation, along with aspects of culture and food safety, is to make fermented food in the classroom, ideally accompanied by creating hypotheses and designing experiments to test them, e.g. testing what happens if the starter culture is not added (Meléndez, 2019; Verran *et al.*, 2019).

While sugar may serve to preserve certain foods, it is all too often added in excess to attract consumers, impacting on health in many ways, e.g. tooth decay, obesity and diabetes. In particular, controversy surrounds the use of high‐fructose corn syrup (HFCS), a sweetener commonly added to processed foods and manufactured by a multistage process driven by microbial enzymes (Crabb and Shetty, 1999). Discussions can consider the Cold War as a spur for the development of HFCS in the United States due to reduced sugar cane supplies; the types of enzymes involved and why/how some are sourced from extremophiles (particularly thermophiles); and, since fructose intolerance/malabsorption is relatively common, issues of intestinal discomfort, the gut microbiome and food choice.

The carbon footprint associated with food, particularly meat production, has led to renewed discussions about alternative food sources, including microbe‐based food, feed and supplements. The thought of eating microbes may initially be viewed with trepidation by many children, until they discover those microbes and microbial products that they may be consuming already, such as mushrooms and those in yoghurt, cheese, salt, fermented foods, yeast extract, *Quorn* or *Spirulina*. Students should be encouraged to consider which microbes may be safe to eat, what biopolymers microbes are made of, and whether they could satisfy animal nutritional requirements. The economics and energy requirements associated with cultivating microbes should be a prime consideration, which may lead to discussions about the diversity of microbial metabolic processes and their capacity to grow using cheap feedstocks. In this respect, the possible use of autotrophic, aerobic, hydrogen‐oxidizing bacteria to generate protein as a food supplement provides and informative case study (Sillman *et al.*, 2019).

### Agricultural, horticultural and aquacultural operations

#### Context

Agriculture uses vast swathes of land, provides jobs and livelihoods for many millions of people and, together with aquaculture, produces most of the food needed for the survival and growth of the world’s human population, our working animals and companion pets, as well as renewable materials for direct use (e.g. for clothing or construction) or conversion to useful chemicals or energy. Food production via aquaculture is increasing rapidly, now supplying more than half of the world’s fish and shellfish for human consumption (FAO, 2016). But, the need to feed a growing human population requires further enhancement of output, through sustainable intensification of agriculture and aquaculture (i.e. increased food production with reduced environmental impact; Godfray and Garnett, 2014; Cavicchioli *et al.*, 2019). Increasing food production in the face of relentless reduction in the available area and quality of agricultural land – the soil crisis – is a mounting challenge. Decision‐making in agricultural policy and practice is a difficult, complex and sometimes contentious undertaking, due to, for example competition between food and renewable production, as well as agricultural practices and global warming‐related extreme weather events causing loss of soils and agricultural acreage. Then, there is the conflict between high yield and sustainability, for example: the use of growth promoters and antibiotics in livestock leading to pollution and the spread of antibiotic resistance; fertilizer run‐off resulting in eutrophication of lakes, rivers and inshore seas; and pesticides polluting soils and water bodies, including those which supply our drinking water, with consequences for non‐target insects (e.g. pollinators and ecosystem engineers) and other animals including humans, plants and microbes.

Plant and animal microbiomes contribute enormously to the health of their hosts and hence to the yields of crop plants and food animals in agriculture and aquaculture (Mueller and Sachs 2015; Martin *et al.*, 2017; Trivedi *et al.*, 2017). Furthermore, agricultural soils need diverse and active microbial communities to maintain soil structure, outcompete pathogens, increase nutrient availability, provide resistance to stress and generally promote plant growth (Reid and Greene, 2013; McNear, 2013; Lee *et al.*, 2020). Similarly, diverse microbial communities are required to maintain healthy water bodies used for aquaculture (Dittmann *et al.*, 2017).

Despite their crucial influence on animal and plant productivity, microbial contributions are usually lacking from considerations of agriculture and aquaculture policy and practice, and the resulting decisions, so the base of available evidence of such decisions is incomplete and hence flawed. Microbiology literacy acquired through childhood education is essential for development of evidence‐based policies and decisions in agriculture and aquaculture relating to feeding world citizens, conserving productive soils, reducing environmental pollution and eutrophication and minimizing oxygen depletion of waters and its resulting lethal consequences for aquatic wildlife and thus sustainability. Therefore, excursions to agriculture, horticulture or aquaculture operations can be both fascinating for children to personally experience the origins of the food that appears on their plates, and important for increasing awareness of global grand challenges and their microbiology components.

#### Suggestions

Below, we outline some of the ways in which microbes influence the environment and food production, which can be explored by contacting the relevant landowners to arrange visits to farms (arable, livestock, shellfish and fish), forests and horticultural centres. Many universities, agencies and research centres (see Section 5) may be visited to obtain a more intimate understanding of the underlying microbiology. There are also industries that supply the agriculture and aquaculture sectors, providing microbial cultures that are added to soils to promote the health and growth of plants (biofertilizers), and to aquaculture waters as probiotics or to recycle excess nutrients from faeces and dead organisms (Martínez‐Córdova *et al.*, 2015). Finally, school or community farms provide an excellent way for children to become more invested in soils, plants, animals, including (shell)fish, and their microbiomes. Where space and cost are at a premium, edible fungi can be grown by inoculating bags of compost with spores. This can also serve as the basis of an agricultural experiment, for example by testing the effect of moisture and temperature on the rate of fruiting body formation.

**Fig. 5:**
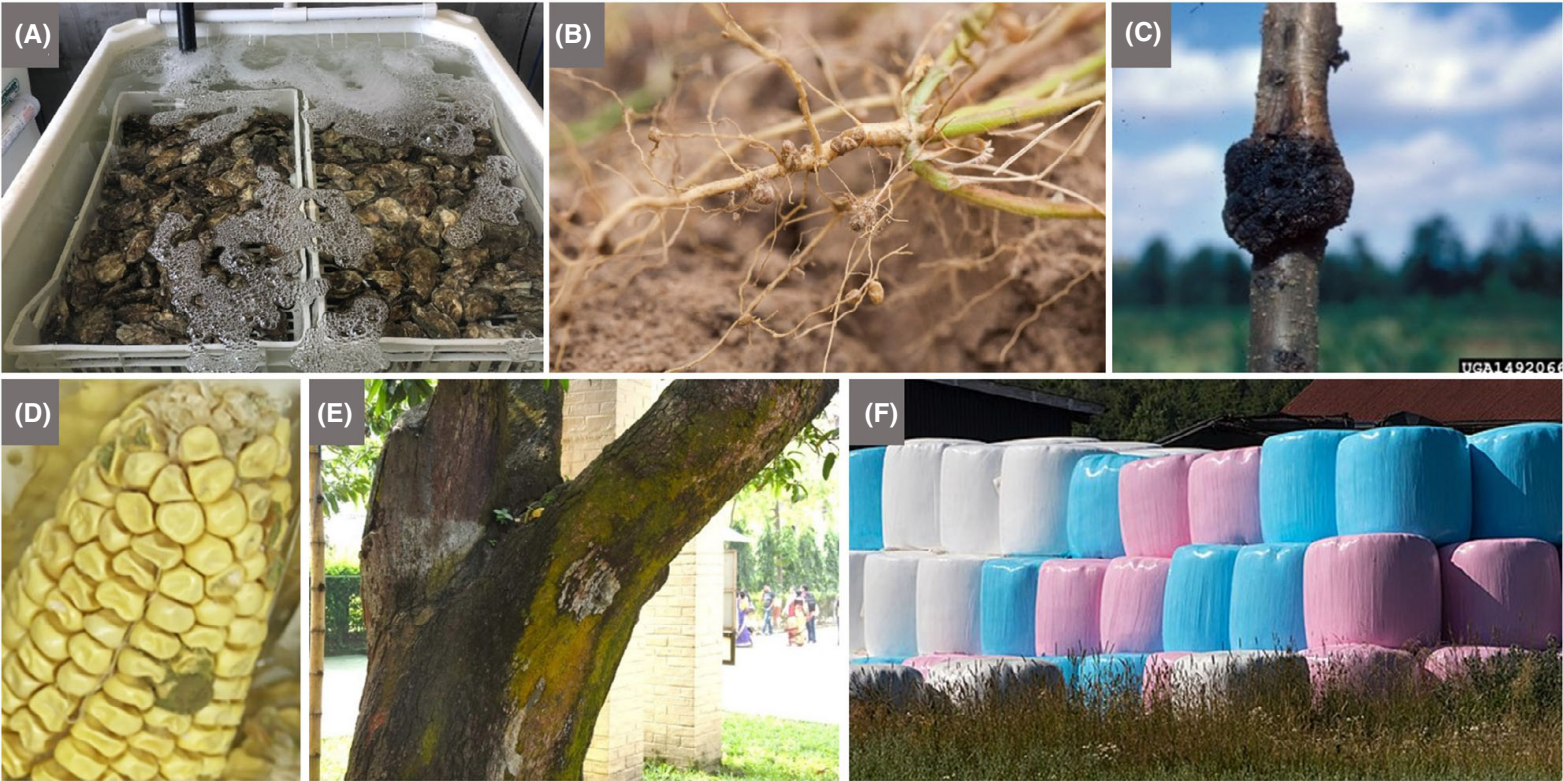
The beneficial and deleterious effects of microbes can be investigated on an excursion to a farm, horticulture centre or aquaculture facility A. Depuration tanks holding Pacific rock oysters (*Magallana gigas*, formerly *Crassostrea gigas*) harvested from the Colne Estuary, UK, by Colchester Oyster Fisheries. The incoming water is treated with ultraviolet light to reduce its microbial load. This process provides time for the filter‐feeding oysters to flush out any pathogens that they may have accumulated while they were in the estuarine waters, before being sold. In addition to providing a potentially sustainable source of protein, bivalves are important ecosystem engineers. For example, they help to ameliorate eutrophication directly by taking up nitrogen and phosphorus for shell and tissue growth and indirectly by creating anoxic sites that encourage denitrifying bacteria that convert soluble nitrate to nitrogen gas (van der Schatte Olivier *et al.*, 2018). B. Nodules housing nitrogen‐fixing bacteria on the roots of southern pea, also known as cowpea (*Vigna unguiculata*), an important food crop in the semiarid tropics. C. Peach tree (*Prunus persica*) with crown gall, a cancerous growth caused by the bacterial species *Agrobacterium tumefaciens*. D. *Aspergillus flavus,* an opportunistic pathogenic fungus, growing on maize. *A. flavus* is the main producer of aflatoxin B1, the most carcinogenic mycotoxin yet known (Gasperini *et al.*, 2019). E. Visible microbiota on a 50‐year‐old mango tree (Kolkata, India). The various colours on the bark largely reflect different microbial communities (e.g. algae, fungi and lichens) colonizing those habitats to which they are best adapted. Key factors that influence microbial distribution include exposure to the sun, availability of water and secretions from the tree. Thus, trees present a living illustration of the microbial biosphere across a series of environmental gradients. F. Silage bales for year‐round animal feed are wrapped in plastic (pink and blue wrappers were used to raise awareness of breast cancer and prostate cancer, respectively) at Gåseberg Sheep Farm, Lysekil Municipality, Sweden. Before wrapping the hay in plastic, its moisture content is optimized and then checked regularly to maintain ideal conditions for lactic acid fermentation while diminishing the risk of spoilage. The consumption of oxygen by aerobic microbes, followed by the production of acidic conditions by lactic acid bacteria (as part of a diverse community of microbes), minimizes the growth of spoilage organisms. Lübeck and Lübeck (2019) provide a clear review of how lactic acid bacteria and other microbes convert green crops (grasses, legumes and the green part of crops like carrots) into a range of agricultural feed products. *Photograph A by Alex Shakspeare, B by Dave Whitinger released under the GNU Free Documentation Licence, C by University of Georgia Plant Pathology, University of Georgia, Bugwood.org, licensed under a Creative Commons Attribution 3.0 Licence, D by Alessandra Marcon Gasperini, E by John E. Hallsworth and F by W.carter under the Creative Commons CC0 1.0 Universal Public Domain Dedication.*

- Arable farms, mushroom growers and horticulture facilities, to explore:
  ◦ Nitrogen fixation: rhizobial root nodules on legumes (Fig. 5); nodules on trees commonly used in agroforestry and land reclamation, such as *Casuarina* species, formed by the actinobacterial genus *Frankia*; and cyanobacteria associated with *Azolla* water fern in rice paddies
  ◦ Mycorrhiza and nutrient exchange: arbuscular mycorrhizal fungi and ectomycorrhizal fungi
  ◦ Biofertilizers
  ◦ Nitrification inhibitors to restrict N loss and nitrate run‐off to surrounding waters
  ◦ Microbes as biological control agents, e.g. *Bacillus thuringiensis* (Bt) and other microbes that kill insect pests, nematode‐trapping fungi
  ◦ Plant genetic engineering (and the application of the bacterial agent responsible for crown gall disease, *Agrobacterium tumefaciens;* Fig. 5)
  ◦ Retting of flax to make linen
  ◦ Identifying and protecting against plant pathogens, crop‐spoilage agents and their associated toxins (Fig. 5)
  ◦ Mushroom cultivation: substrate preparation (manure fermentation/composting), spawn production and fruiting body production
- Livestock farms, to explore:
  ◦ Silage: the role of lactic acid bacteria in fermentations to extend fodder life and nutritional quality (Fig. 5)
  ◦ Feed supplements, such as amino acids and antibiotics synthesized by microbes
  ◦ Phytases: enzymes added to animal feed to enhance the bioavailability of phosphorous
  ◦ Animal faeces as proxies of the complex microbial bioreactors of the gastrointestinal tract, including the rumen microbiome, where microbes assist digestion of cellulose‐rich feed (and, in the process, produce much methane)
  ◦ Animal waste and the circular economy: biogas production and spreading manure, slurry, digestate from anaerobic digestion, which recycles N and P fertilizers and enriches microbial diversity of soils
  ◦ Methods to reduce the microbial load of foods, e.g. by pasteurization of milk
  ◦ Identifying and protecting against livestock pathogens
- Fish and shellfish farms, to explore:
  ◦ Microbially derived polyunsaturated fatty acids to enrich live feed (e.g. rotifers and *Artemia*)
  ◦ Measures to improve disease resistance by adding probiotic/biocontrol cultures
  ◦ Supplementing fish and shellfish diet with prebiotics such as short‐chain fatty acids and the microbial storage compound, polyhydroxyalkanoate, to improve the health of the gastrointestinal tract community and protect against pathogens (e.g. Defoirdt *et al.*, 2009)
  ◦ Measures to improve water quality for aquaculture, e.g. by microbiological removal of hydrogen sulfide and ammonia
  ◦ Methods to reduce the microbial load from filter‐feeding molluscs, e.g. by depuration (Fig. 5)
  ◦ Identifying and protecting against pathogens of fish and shellfish

#### Some discussion topics

Numerous learning opportunities arise from investigating microbes in agriculture. For example, root nodules on legumes are visible manifestations of invisible microbes mediating an exceptionally important activity, namely nitrogen fixation by symbiotic Bacteria that provide plants with nitrogen in a form that can be used, e.g., for the production of proteins, thereby facilitating plant growth (Fig. 5). Similarly, observations of fungal hyphae in close association with plant roots may serve to introduce the roles of mycorrhizal fungi. Teacher‐guided discovery by children of root nodules and plant‐associated fungi can elicit discussions about plant–microbe symbioses in the rhizosphere – the intimate microbiome interface between the plant root and its soil medium – where crucial microbially driven physiological processes unfold that determine the availability of: a) nutrients, like nitrogen and phosphorus, essential to plant growth, b) hormones that stimulate plant growth, and c) pathogen inhibitors that suppress microbes causing root disease. Root microbiomes have also been shown to protect plants against drought, extreme temperatures and other stresses.

Visits to farms and garden centres can be linked to education about land use and soil as a resource, the ‘soil crisis’ (Koch *et al.*, 2013), and the beneficial roles of microbes in forming/maintaining healthy and productive soil. For example, in addition to contributing to soil formation by rock weathering (Napieralski *et al.*, 2019) and soil fertility, some soil microbes ‘glue’ together soil particles and reduce soil loss by wind and rain, and others degrade pollutants, including agricultural pesticides. The processing of crops grown as sources of renewable energy or chemical production often involves microbial activities, on‐site or off‐site, which can be discussed. The concept of the circular economy can be introduced and issues explored, such as spreading of animal waste on agricultural soils and the potential to unintentionally spread antibiotic‐resistant microbes and pathogens that may also infect humans (Berendonk *et al.*, 2015). Plant diseases caused by rusts, blights, wilts, galls, and their visible manifestations, on crop and non‐crop plants, can be stimuli for discussions about the causes of disease and different means of prevention or treatment, as well as their role in contributing to famine and influencing human history. A visible infestation of crops by aphids can be used to introduce the topic of the insect’s dependence (and *vice versa*) on its endosymbiotic Bacteria, *Buchnera* species, and more broadly, it can serve to introduce the topic of microbial control agents in agriculture, such as the insect‐killing bacterium *Bacillus thuringiensis* (Bt), used primarily to kill larvae of Lepidoptera (Bravo *et al.*, 2011; Engel and Moran, 2013). Cattle farmers can explain the problem of bovine tuberculosis, which can lead to microbiology‐informed debate about disease transmission and the pros and cons of badger culling.

And, not to forget, soils are a major repository of microbial diversity and hence the major source of microbiome diversity maintenance (in humans and other life forms), which is crucial for the health of most species in the biosphere (‘Let them eat dirt’; Finlay and Arrieta, 2016), which is also an interesting topic for children to explore.

A visit to a marine or freshwater aquaculture facility, quite apart from the interest in seeing the process of fish/mollusc/crustacean rearing, and the different developmental stages, provides an excellent platform to explore food webs (e.g. phytoplankton (and their associated beneficial heterotrophic microbes) – zooplankton – fish) and thus the interconnectedness of life, as well as the problems of the use of growth promoters and nutrient/faecal pollution that can lead to eutrophication and oxygen limitation of water bodies.

### Environmental and industrial biotechnology sectors

#### Context

Microbes are central to the environmental and industrial biotechnology sectors, which contribute a burgeoning array of products and processes for sustainable economic growth (Timmis *et al.*, 2017). These range from municipal wastewater treatment plants to biotechnology start‐ups, and include long‐running chemical giants that have switched to using biological processes. These enterprises can be identified through local knowledge, directories, internet searches, asking school children and colleagues where family members work and contacting local universities and research centres.

**Fig. 6:**
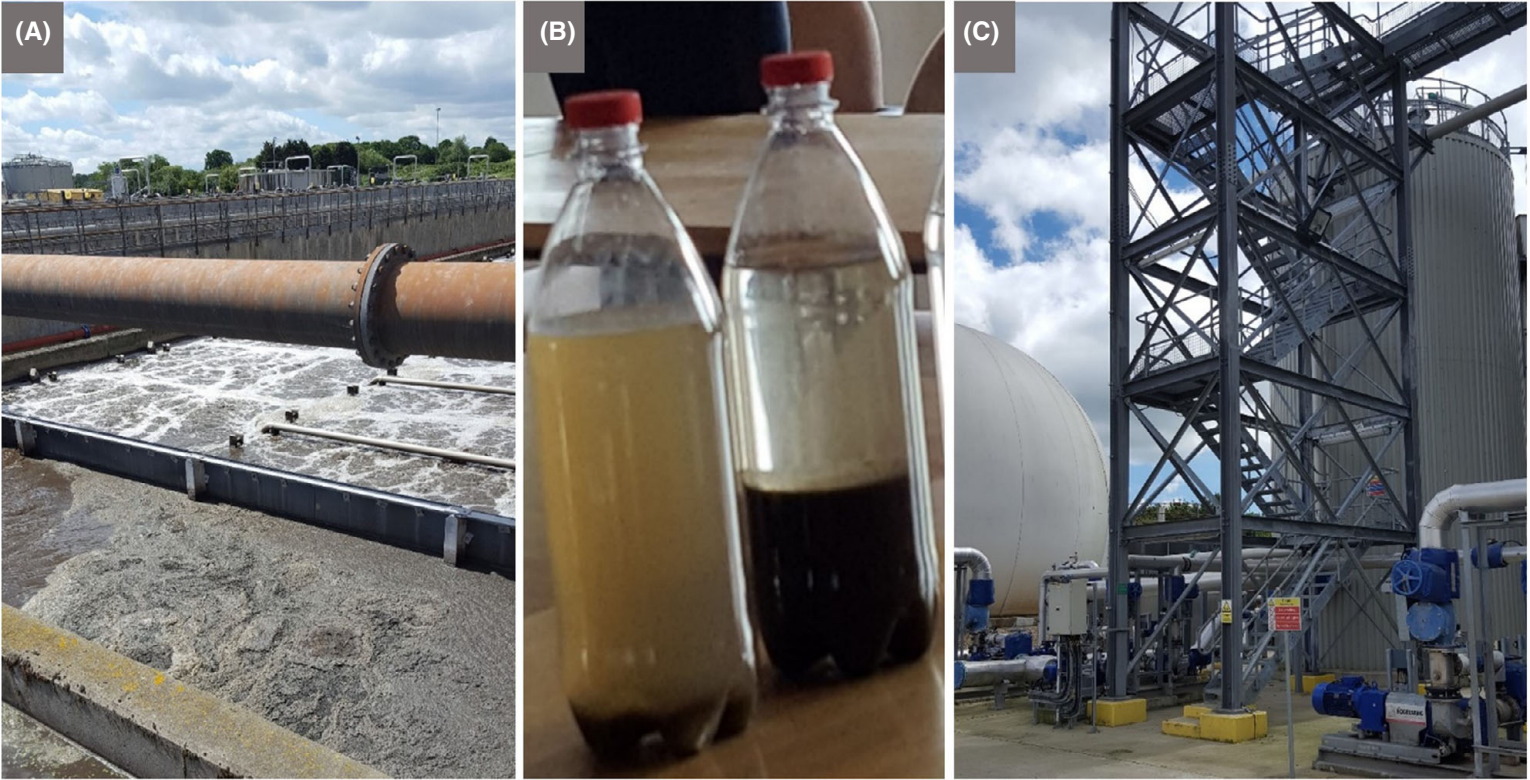
Wastewater treatment plants as an opportunity to see microbes as the ultimate recyclers A. Colchester wastewater recycling plant run by Anglian Water. The image shows part of the activated sludge treatment, whereby flocs are encouraged to form, due to the activity of myriad microbes, including the extracellular polymer‐producing bacterial species *Zoogloea ramigera*. The organic microbe‐rich flocs are allowed to settle (see B): some will be recycled to seed the activated sludge tanks, and the rest can be used to make biogas (see C). B. The right‐hand bottle illustrates how activated sludge settles, thus separating the solids and leaving behind reasonably clear liquid with reduced organic load. The left‐hand bottle shows incomplete settling due to a suboptimal activated‐sludge microbial community. C. The upstream part of the anaerobic digestion facility at Colchester wastewater recycling plant. The sludge from the plant and surrounding locales is subjected to heating, pasteurization and hydrolysis, before the anaerobic digestion step, driven by methanogenic Archaea. The resultant methane is used to generate electricity and can be stored in the biogas dome shown on the left. *Photographs by Terry McGenity.*

Societal responsibility necessitates that everyone learns what happens to the wastes we produce, so that we transit from an *out‐of‐sight, out‐of‐mind* mentality when we flush the toilet or empty rubbish into the dustbin, to an awareness of the processes, needs and costs of what is thereby actioned. An excursion to a wastewater treatment plant/recycling facility can provide a particularly interesting and wide‐ranging microbiological experience, and almost every town has one (Fig. 6). Water companies are often accommodating to visits, and they may have specialized outreach professionals/microbiologists available. The olfactory component ensures a memorable visit, and the change in smell while progressing through the treatment plant demonstrates that the underpinning microbiological processes are working. Wastewater treatment eliminates much of the waste we produce and channel into the wastewater disposal system. It also removes a major fraction of the pathogens present in wastewater and thereby enormously reduces disease transmission and our infection burden. Currently, wastewater treatment focuses not only on waste destruction but increasingly on recovery of resources (notably the macronutrients nitrogen and phosphorus, but potentially gold and other precious metals), the recuperation of energy in the form of methane gas from anaerobic digesters and the creation of added‐value products like biopolymers, and hence plays a central role in a number of facets of the circular economy and societal development. Industrial wastewater treatment plants can additionally demonstrate the destruction of wastes, such as polluting chemicals, produced by commercial operations.

#### Suggestions

- Wastewater/sewage treatment/recycling facilities: activated sludge, trickling filters, anaerobic digesters producing biogas and digestate, etc.; also low‐tech alternatives, e.g. septic tanks, simple bioreactors, constructed wetlands, composting facilities, nutrient removal/recovery and creation of added‐value products (Morgan‐Sagastume *et al.*, 2010; Nielsen, 2017; Vriens *et al.*, 2017) (Fig. 6)
- Drinking water supply companies: (toxic) algal/cyanobacterial growth in reservoirs, pollution of groundwater, filtering methods and disinfection methods
- Water quality testing laboratories: biological oxygen demand, ATP test, testing by cultivation or molecular biology methods, e.g. for coliforms, *Escherichia coli, Legionella pneumophila*, *Cryptosporidium* spp. and norovirus
- Waste composting companies and associated testing for greenhouse gas emissions and bioaerosol formation
- Landfill sites and how leachates are treated
- Companies developing/employing biofiltration methods to remove volatile organic compounds, sulfide, etc.
- Companies involved in bioremediation/detoxification of environmental pollutants, such as petroleum, restaurant fats, oils and grease, chlorinated solvents, explosives and heavy metals
- Fossil fuel (petroleum, gas and coal) industries and companies providing services to them
- Companies using microbes to leach/mine metals from low‐grade ores
- Companies employing microbiological methods to capture carbon
- Companies developing environmental biomonitoring tools, e.g. biosensors, Microtox assay, microalgal indicators and microbial community indices
- Enzyme producers: research laboratories, pilot‐scale testing facilities, production facilities and quality control laboratories
- Manufacturers of bio‐based platform chemicals: e.g. itaconic acid, propane‐2‐diol, isoprene and acrylamide
- Manufacturers of tools for molecular biology: e.g. green fluorescent protein, enzymes, cloning vectors and CRISPR‐Cas9 for genome editing
- Manufacturers of scents, flavourings, pigments, compatible solutes for healthcare and well‐being products (e.g. ectoine), etc.
- Manufacturers of biopolymers/bioplastics/biosurfactants: e.g. microbial cellulose, polyhydroxyalkanoates, polylactate and xanthan gum
- Companies using fungi or bacterial cellulose mats to make furniture, fabrics, packaging, clothing dyes, etc. (see *Microbial Biotechnology* 12: 4 special issue on microbial cellulose)
- Industries that use microbial enzymes, including paper, leather, detergent and food‐processing sectors
- Biofuel production plants (bioethanol, biobutanol, microbial hydrocarbons, biodiesel and biogas)
- Agrochemical companies: research laboratories, production facilities, quality control laboratories, e.g. involved in developing new pesticides based on microbial metabolites
- Biofoundries
- Government institutes and regulatory agencies

#### Some discussion topics

Diverse topics of microbiology in action can be addressed during a visit to a wastewater treatment plant, depending on the age group and interests of the students and teachers, such as floc formation/biofilms, nutrient cycling, the water cycle, aerobic versus anaerobic processes, pathogens, antimicrobial resistance, anaerobic digestion and the formation of biogas and digestate (Fig. 6). A close look at trickling filter beds can reveal a zoo of micro‐ and macro‐organisms contributing to the wastewater treatment process, leading to discussions about food webs. Protozoa and microbial metazoa grazing on Bacteria and Archaea make for good viewing under the microscope. Even without a microscope, children can see chironomid midge larvae which feed on the microbial biofilms, thereby keeping them continually growing and optimally active, as well as preventing the filters from becoming clogged. At landfill sites, children can explore microbial aspects of: the sources of the smell and how microbes can also be used to remove smelly volatile compounds, differential rates of degradation of different materials, biodegradable plastics, pollution from leachate and microbial remediation, coastal erosion and the impact of landfill waste entering the sea, generation and harnessing of methane from landfill. And, since sites for landfill compete with other uses, like agriculture and housing, the need to reduce landfill expansion and the key issues of separation, recycling, resource recovery and microbial involvement in these provide discussion topics for lifestyle decision‐making, which should encourage future generations to value and conserve resources.

When visiting petroleum companies or their field operations, or those organizations providing services to the petroleum industry, children can be taught about the role of microbes in cleaning up oils spills, including gasoline released from leaking storage tanks at petrol stations, as well as a range of other microbially driven processes, such as: in‐situ depletion of sulfide in oil reservoirs by encouraging nitrate‐reducing microbes, biodesulfurization of crude oil and coal, microbially enhanced oil recovery and prevention of microbially induced corrosion of metal structures. The impact of fossil fuel industries on climate and the extent to which petroleum‐based fuels and other products should still have a place in society are pertinent topics for discussion. However, when considering alternative energy and chemical sources, e.g. when visiting bioethanol or other biofuel plants, in addition to learning about the underlying biological process, the sources of the feedstock should be considered and the environmental and societal cost of its production deliberated, i.e. the *fuel‐versus‐food* debate. Then, the children can consider why it is preferable to produce sugar (as precursors for some biofuels) from alternative sources, particularly waste lignocellulosic material (e.g. waste paper, straw), followed by discussion of the types of microbes that degrade lignin and cellulose, where they can be found and how to harness their activities.

### Health and medical sectors

#### Context

Children, for reasons of *inter alia* immunological naivety, overly hygienic upbringing in their early years, and classroom confinement, are particularly susceptible to infections. The physical and mental suffering caused by microbial disease affects children considerably, not least because of school absences and the stress of catch‐up, and frequently motivates them to want to learn more about, and sometimes to choose a career in, microbiology or medicine. Poorly evidenced propaganda about supposed negative effects of vaccination has led to the re‐emergence of some well‐contained, or almost eliminated, microbial diseases (e.g. measles and polio). Outbreaks of pathogens causing diseases characterized by high mortality rates (e.g. Ebola virus) or high infectivity enabling epidemic or pandemic spread (e.g. SARS‐CoV‐2 causing COVID‐19) occur regularly. Crucially, the global increase in antimicrobial resistance in previously treatable pathogens means that many infections have now become life‐threatening (Timmis *et al.*, 2019) and are predicted to become a major cause of global morbidity and mortality in the future.

**Fig. 7:**
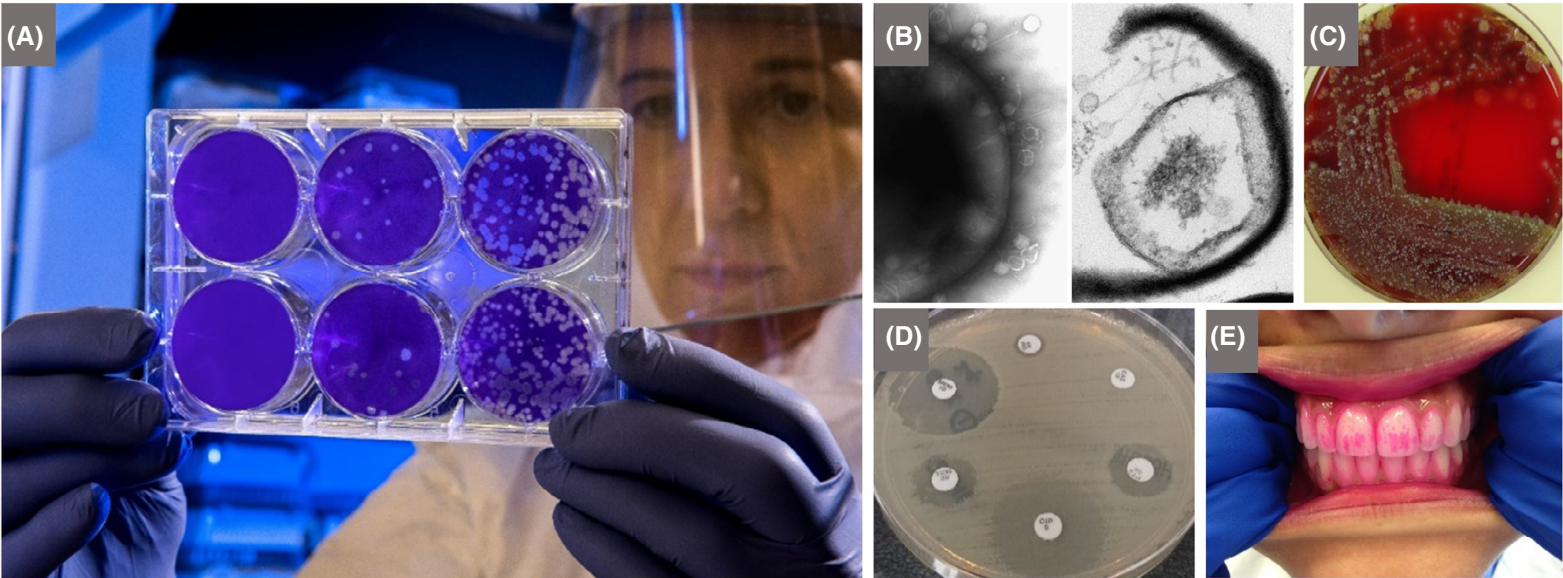
Diagnostic and public health laboratories allow students to investigate different ways of growing, detecting and viewing microbes, and learning about treatment of pathogens A. Viral plaque assay to quantify influenza virus. Dilutions of the sample are added to a human cell line and incubated. The purple stain shows cells that are not infected with the virus, while clear zones (plaques) indicate viral infection and cell lysis. B. Some laboratories may perform electron microscopy, giving fascinating insights into the shape, size and ultrastructure of Bacteria and viruses. These images are transmission electron micrographs of the bacterium, *Clostridium difficile,* being attacked by bacteriophages. Left, surface of an intact cell of *C. difficile,* showing large numbers of attached bacteriophages, most with empty hexagonal heads, representing bacteriophages that have injected their DNA into the bacterium. Right, part of a *C. difficile* cell that has burst open due to the proliferation of bacteriophages within, which produce enzymes that lyse the cell membrane. The height of both images is ~ 800 nm. C. Throat swab sample inoculated on a blood agar plate, showing colonies of *Streptococcus pyogenes*, the causative agent of tonsillitis and other diseases, identified by β‐haemolysis (the production of clear zones around bacterial colonies, resulting from lysis of red blood cells by the haemolysin produced by the pathogen). D. *Staphylococcus aureus* antibiotic sensitivity testing using the disc diffusion method. *S. aureus* is an example of a pathogen, frequently acquired in hospital, that has developed resistance to multiple antibiotics. A culture of the bacterium is spread on the agar plate, and discs containing different antibiotics are then placed on the plate. During incubation, a bacterial lawn develops. A zone of clearing in the lawn around a disc indicates that the bacterium is sensitive to the corresponding antibiotic, thereby contributing to diagnosis as well as informing treatment strategies. E. Staining teeth biofilms (plaque) with plaque‐disclosing tablets provides a visual and memorable way to drive home the importance of good dental hygiene to remove plaque and thus prevent dental caries and periodontal disease (note the different meanings of the term ‘plaque’ here and in A). *Photograph A by CDC on Unsplash, B by Stefan Hyman with samples from Martha Clokie, University of Leicester, C by Aurélie Villedieu, D by Selwa Alsam and E is licensed under the Creative Commons Attribution‐ShareAlike 4.0 International licence.*

On the other hand, there are exciting advances linking the human microbiome with well‐being, which promise to lead to new paradigms for diagnosis, prevention and therapy of both physical and mental health conditions originating from microbiome perturbations. The, by now, classical example of perturbed microbiota restoration – the successful use of faecal microbiota transplants to treat recurrent or refractory *Clostridium difficile* infections promoted by antibiotic therapy – is encouraging the assessment of similar interventions for other conditions (Mullish *et al.*, 2018). And bacteriophage therapy, another ecological strategy to deal with bacterial infections, is making a comeback (Brüssow, 2017; Gordillo Altamirano and Barr, 2019; Fig. 7).

Some of the obvious and interesting places to learn about the medical sector may not be practical for school excursions, especially for younger children, e.g. hospitals to learn about hygiene procedures in surgical and intensive care units and the importance of immunosuppression, and primary healthcare clinics to discover diagnostic procedures, what clinical samples reveal, and what tests will be carried out. However, others, especially teaching hospitals, may have educational facilities that can accommodate class excursions, and some primary healthcare clinics may also be amenable to organized visits, so it is worth enquiring. Health clinics and hospitals can be associated with physical and/or emotional discomfort, causing some children to view them with apprehension, so visits in a learning capacity can be very useful to establish familiarity and create a more positive, balanced perception.

Some diagnostic laboratories may be amenable to school visits, and public health agencies often have outreach divisions, and may also allow/encourage visits. Academic centres, learned societies and museums all provide opportunities to learn about the role of microbes in health and disease, and health workers often visit schools to talk about microbiological topics, such as sexually transmitted diseases. Opportunities offered by healthcare‐system outreach programmes, and by the availability of enthusiastic health professionals for outreach activities, can significantly enhance the educational benefits of health‐centric excursions and in‐school follow‐ups.

#### Suggestions

Some organizations for health‐related microbiology‐centric excursions are given below:

- Hospitals
- Clinics
- Public health agencies
- Pathology and diagnostic laboratories, including drug‐resistance testing
- Veterinary laboratories/surgeries
- Pharmaceutical companies: research laboratories, production facilities and quality control laboratories, e.g. involved in new medicines based on microbial metabolites
- Manufacturers of enzymes used in medicine
- Companies developing diagnostic kits for detecting pathogens or their effects

#### Some discussion topics

In order to ensure a balanced narrative, before discussing pathogens and disease, it is helpful to remind students of the ways in which microbes promote health or reduce risk of disease, such as: microbiome steering of immune‐system development in infants, microbiome inhibition of pathogen colonization and reproduction on body surfaces, biotechnological use of microbes and microbial products to create vaccines and therapies, destruction of pathogens by microbial activities in wastewater treatment, preservation of food by fermentation, enhancement of crop yield and degradation of pollutants.

Children enjoy learning about disease and benefit from clear, contextualized images of the relevant causative pathogens under discussion, in terms of their size (e.g. relative to something that the particular age group would recognize), type (e.g. Bacteria, viruses and protozoa) and source/reservoirs of infection. Epidemiological games (e.g. Centre for Disease Control’s *Solve the Outbreak*), incorporating elements of forensic detective work, provide an illuminating way to illustrate how disease can spread.

Opportunities to relate infection dangers to everyday life should be embraced. For instance, discussion of modes of transmission and their prevention is particularly interesting to young children who play outside and have pet animals, and may usefully include transmission by biting insect vectors. Examples include *Borrelia burgdorferi,* a bacterial species that causes Lyme disease and is transmitted by ticks residing in grass, and *Plasmodium,* a protozoal genus with several species that cause malaria, which is transmitted by mosquitoes. Zoonoses – the transmission of infectious agents from reservoir animals to humans – also include *Borrelia,* whose primary hosts are mammals, and *Salmonella* serovars*,* which infect chickens and may be transmitted to us in our food if we do not observe good hygiene and properly cook the meat. And, particularly topical is the genetic evolution of animal pathogens, like HIV, as well as SARS, MERS and other coronaviruses, to become human pathogens (Cui *et al.*, 2019; Brüssow, 2020). All of these topics enable the teacher to discuss relevant practices to help restrict the spread of pathogens, such as washing hands before handling food or eating and after going to the toilet, environmental management (e.g. water treatment for cholera), avoidance (e.g. avoiding physical contacts, like handshaking/hugging, social distancing and quarantining during epidemics like that caused by coronavirus), protection (e.g. spraying with mosquito repellent for malaria and using condoms for sexually transmitted diseases), vaccination and treatments (e.g. whether or not to use antibiotics). These issues can lead to discussion of broader topics, including One Health (see WHO web pages) – public health strategies based on the integrated nature of human, animal and environmental health – which targets the key health‐relevant components in this interactive network. In this context, an important issue for discussion is the spread of antibiotic resistance through the use of antibiotics in farming and aquaculture. Moreover, such personally relevant, and sometimes frightening, issues provide an opportunity to consider the dissemination of misinformation via the internet and thus the importance of the scientific method, ethics and communicating research.

Diagnostic and public‐health laboratories can introduce children to the different levels of pathogenicity and infectivity of pathogens, and thus containment needed for safe handling, as well as the different means of detection, typing and quantification (Fig. 7). Older children can be introduced to the application of molecular methods for detection of pathogens, e.g. using the polymerase chain reaction (PCR), the underpinning of which was the discovery of the thermophilic bacterium, *Thermus aquaticus,* from colourful thermal springs in Yellowstone National Park, which was the source of thermostable DNA polymerase that made PCR feasible. Visits to pharmaceutical companies will focus on the development and manufacture of therapeutic drugs, e.g. for microbial diseases, and the process of drug discovery. Inspiration for many pharmaceuticals is often drawn from nature, and it will help children to value microbes if they recognize that they are the source of much of that inspiration or the products themselves. So, it is useful to make connections with everyday sensations, such as the smell of soil after rain caused by geosmin‐producing *Streptomyces* species, which are the source of a range of antibiotics and other pharmaceuticals. A natural development to this discussion would be to consider the role that such secondary metabolites play in nature.

### Research centres, academic‐guided excursions and children’s involvement in research projects

#### Context

Many schools are in visiting distance of an academic research centre, where children can experience the cutting edge of discovery by a variety of means listed below. Within academia, outreach activities are generally encouraged, and specific outreach days are often hosted. Sound advice to specialists on communicating science to children and the general public is provided by many learned societies and a variety of publications (e.g. Bowater and Yeoman, 2012; Westenberg, 2016; Illingworth and Prokop, 2017). Academic centres have an advantage over many commercial or industrial settings in that they are likely to have laboratories that can accommodate demonstrations, hands‐on experiments and bioinformatics. They can also be a good source of contacts to relevant industry, e.g. via their technology/science parks and research connections. In addition, visits to an academic centre can often facilitate choices of study topics and create contacts helpful for future tertiary education and internships.

Involving children directly in research activities, for example through joining microbiologists undertaking fieldwork, provides first‐hand experience of what being a microbiologist is like. As an example of one exercise organized by the Danish Technical University and supported by industrial sponsors, school children collected samples to isolate lactic acid bacteria and made headline news with the discovery of 10 new species. Such activities, and larger‐scale citizen‐science or mass‐experiment projects, give direct experience of the scientific method, including hypothesis setting, experimental design and problem solving. Importantly, this will promote enthusiasm for microbiological research, and the principles learned can be applied to tackle complex problems encountered in later life.

Schools can be involved in a range of citizen‐science activities, many of which do not require a visit to a nearby academic centre, as instructions and materials may be provided or otherwise easily obtained. The ‘Small World Initiative’, developed at Yale University as a citizen‐science programme in which students isolate microbes from their local environment and identify antibiotics produced by them, has grown into a wider research programme (Davis *et al.*, 2017). It has also been adapted in several other countries, e.g. to encourage the involvement of school children and include aspects of service learning (Valderrama *et al.*, 2018), i.e. combining learning objectives with community service (Webb, 2017). Another example of service learning is provided by Rutgers University graduate students, who run a mobile laboratory, from which, among other activities, they introduce school children to the spread of microbial infections (Irizarry‐Barreto *et al.*, 2018). DTU Bioengineering also has an effective model, whereby students run an organization named the Biotech Academy, which provides outreach and teaching in schools.

The long‐running initiative called ‘Discover the Microbes Within! The *Wolbachia* Project’ enables students to see what it is like to be a scientist by participating in research. It is premised on the facts that arthropods represent about 85% of all animal species, and the bacterial endosymbionts, *Wolbachia* spp., occur in at least 40% of all arthropod species. Therefore, immersion microbiology in this symbiotic world is accessible to all students and classrooms across the world. This project allows a wide selection of arthropods and habitats to be investigated and involves children in the search for *Wolbachia* symbionts and their bacteriophages.

#### Suggestions

Learned societies, charities and foundations fund and host events, and support and mobilize scientists, with the remit of engaging schools and the public in science. Teachers and pupils can take advantage of these events by being members themselves and/or subscribing to the relevant mailing lists. Nearly all academic centres, and/or the departments within them, have personnel responsible for outreach activities, who can provide information about what they can offer from among the following:

- Showcasing research activities, including case studies of research with broad societal impact, as part of a school visits, courses or public open days
- Providing links to associated science/technology parks, giving insight into spin‐out companies and small‐to‐medium‐size enterprises that are carrying out early‐stage research and development in many of the areas listed in previous sections
- Organizing and/or supporting field‐based projects, hands‐on wet laboratory and bioinformatics experiments (where appropriate, with a follow‐up visit to the school class to present the results and discuss interpretations)
- Providing seminars and interviews remotely (e.g. webinars and interviews by video conference), especially in response to high‐impact scientific publications
- Preparing and providing teaching resources (e.g. websites, games and videos) and loaning equipment (see ‘Importing excursions into school, and virtual excursions’ below)
- Advising about activities associated with microbiological promotional days/weeks (e.g. International Microorganism Day, UK Fungus Day, World Microbiome Day and World Antibiotic Awareness Week)
- Supporting large‐scale projects with the public as sample collectors, observers, analysts, experimenters and/or direct subjects of enquiry, e.g.:
  ◦ American Gut (Debelius *et al.*, 2016; McDonald *et al.*, 2018)
  ◦ Belly Button Biodiversity (Hulcr *et al.*, 2012)
  ◦ Wild Life of Our Homes (Barberán *et al.*, 2015)
  ◦ Big Compost Experiment (run by University College London)
  ◦ Tea Bag Index, measuring microbial biodegradation (Keuskamp *et al.*, 2013)
  ◦ Discover the Microbes Within! The *Wolbachia* Project (Bordenstein *et al.*, 2010)
  ◦ Small World Initiative (Davis *et al.*, 2017)
  ◦ Tiny Earth (run by University of Wisconsin‐Madison)
  ◦ The Microcosmos community of Foldscope users (Cybulski *et al.*, 2014; see Section 6)
  ◦ Foldit (Cooper *et al.*, 2010)
- Supporting projects that benefit from expert advice, primarily for students in higher education, but also open to older school children, e.g.:
  ◦ Ocean Sampling Day (Kopf *et al.*, 2015; Schnetzer *et al.*, 2016)
  ◦ International Genetically Engineered Machine (iGEM) Competition (Smolke, 2009)

#### Some discussion topics

**Fig. 8:**
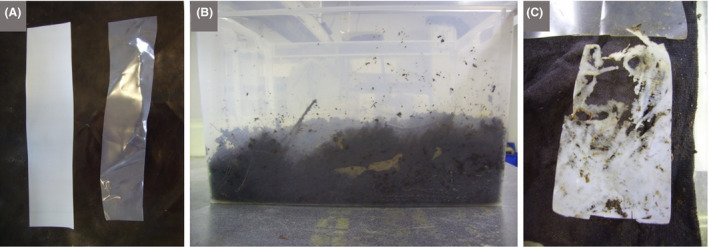
Bioplastic degradation in compost A. Strips (20.8 x 4.5 cm) of polyhydroxybutyrate (left), a biodegradable plastic that can be made by many Bacteria and Archaea (and which serves the role of a carbon reserve in nature), and polypropylene (right), a non‐biodegradable plastic derived from petroleum. B. Garden compost into which the strips were placed, after securing them in nylon tights, which served as ‘litter bags’. C. The polyhydroxybutyrate (below) shows clear signs of biodegradation after 15 days incubation, whereas the polypropylene (above) remains unchanged. *Photographs by Terry McGenity.*

Points to discuss will depend on the research area presented and can be guided by the microbiology expert, and/or ideas should be available on the relevant websites. For example, the ‘Big Compost Experiment’ invites the public to select bioplastic packaging and test their biodegradation, which encourages learning about the chemistry and (micro)biology of different types of bioplastics as well as the composting process (Fig. 8). The discussion could readily be extended to include the wider issue of plastic pollution as outlined in the next section.

### Field excursions

#### Context

An advantage of field excursions is that they can take place in accessible local environments, including urban sites and the school grounds, thereby reducing cost, limiting disruption to timetabled classes and allowing repeat visits (Howarth and Slingsby, 2004). And, although the individual microbial agents underlying the activity to be experienced cannot be seen with the naked eye, clusters/networks of cells, biofilms and slimes may be visible, and the activities themselves can be experienced by the senses, visually, and in some cases, by touch, smell, taste (for known foods!) or sound (see the Discussion).

Children are thrilled to discover previously invisible animate beings and inanimate objects, and are captivated by new microscopic worlds in the samples they have taken. Thus, we stress the importance for each school to have at least one microscope. However, a child’s curiosity may dwindle on the walk from the field site to the classroom or in the queue for the microscope, which makes portable and affordable tools like the *Foldscope* extremely valuable for microbiological excursions. By assembling their personal, or team, *Foldscope* beforehand, children appreciate the technology behind the optical microscope. Moreover, the sense of ownership of their *Foldscope*, together with the ability to view their samples immediately, will enhance children’s enjoyment and sense of discovery. The mantra ‘small is beautiful’ translates into reality as invisible microbes are revealed, become tangible, enter the memory of the child, to be automatically recalled and linked to a relevant microbial habitat and/or activity. Then, sharing their achievements and experiences with the global *Foldscope* community is fun and reinforces the interest and reward of their studies: they become active members of a worldwide network of discovery of the microscopic unknown!

In some situations, it may be possible for teachers to incorporate simple field experiments into excursions, involving observations and perturbations, with or without simple measuring devices. This has the virtue of extending class experiments to field experiments and revealing the essential link between the two in obtaining a realistic picture of microbial functioning and contribution to environmental processes.

A wide range of microbial manifestations is explored in the wonderful book ‘A Field Guide to Bacteria’ (Dyer, 2003) and the excellent website ‘Exploring the Invisible’. A few ideas out of hundreds of possibilities are presented here.

**Fig. 9:**
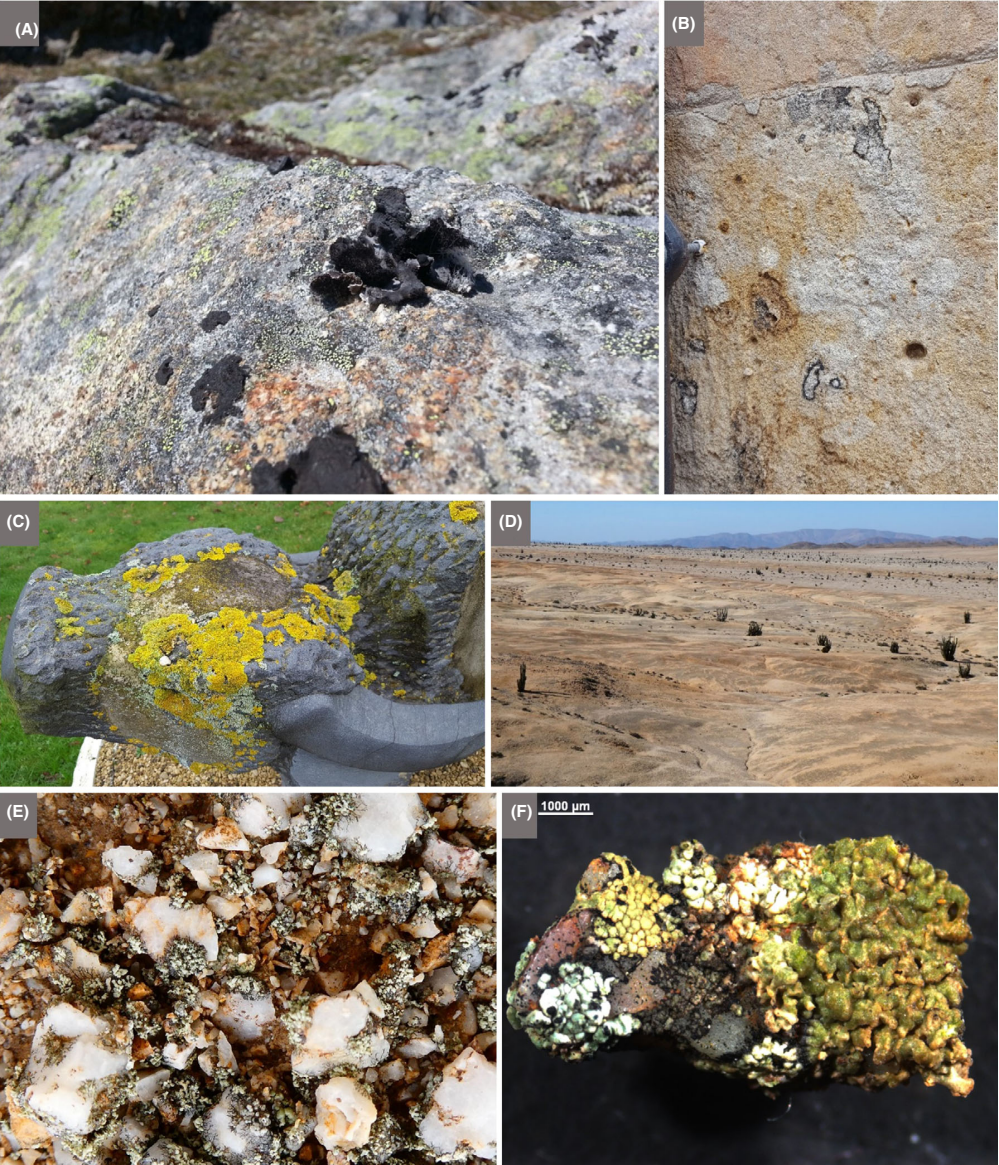
Living skins on rocks A. Diverse lichens, including black foliose forms, on a rock in western Greenland. B. Lichens and fungi, including black ring forms on a sandstone building, which together with microbial communities, contribute to the biodeterioration of Villamayor Sandstone, the main building stone of the UNESCO World Heritage city of Salamanca. The field of view is ~ 20 x 20 cm. C. Lichens on a stone cow, Milton Keynes, UK. D. The seemingly barren landscape of the National Park, Pan de Azùcar, of the coastal Atacama Desert is covered by tiny, white stones that have a diameter of ~ 6 mm (termed *grit*). The black patterns are caused by lichens, cyanobacteria, green algae and fungi that colonize the surface and inner structures of the polycrystalline grits. These organisms also concatenate the single grits forming biological soil crusts (termed *grit crust;* Jung *et al.*, 2020). E. Close‐up of the grit crust after a fog event. Mainly lichens in their wetted stage are visible growing attached to grit stones. F. Single grit stone covered by various lichens together with microfungi (mainly *Lichenothelia*). Many examples of microbes as part of the urban landscape can be viewed at ‘This is Microgeography’ website. *Photographs A–C by Terry McGenity and D‐F by Patrick Jung.*

Microbes and their activities are often more obvious than we realize, and the urban environment with its building stones/materials, wooden structures, monuments and cemeteries provides opportunities to explore the roles of microbes in everyday life as architects of both construction and decay (see ‘This is Microgeography’ website and Fig. 9 for example). Microbial builders include species used to repair monuments by facilitating calcium carbonate cement formation (Gao *et al.*, 2019). It has also been proposed that the extracellular polymeric substances (polysaccharide‐rich slime) produced by microbes degrading cellulose serve as a binder in the construction of mud buildings (Vissac *et al.*, 2017). No surface is free of microbes, and microbial colonies may be visible on buildings, headstones, monuments and rocks in the form of melanized fungi, green algae, multicoloured lichens and microbially formed minerals (manganese and iron oxides, and calcium oxalate) on stone, mortar, plaster or stucco (Gadd, 2017; Fig. 9). Such biofilms, crusts and patinas may lead to deterioration (e.g. via acid‐producing Bacteria, Archaea and fungi) or protection (e.g. by hydrophobic biofilms keeping out water) (Gadd, 2017). Thus, everyday stone objects provide the basis for visualizing microbes, their interactions and the effects of their various activities.

**Fig. 10:**
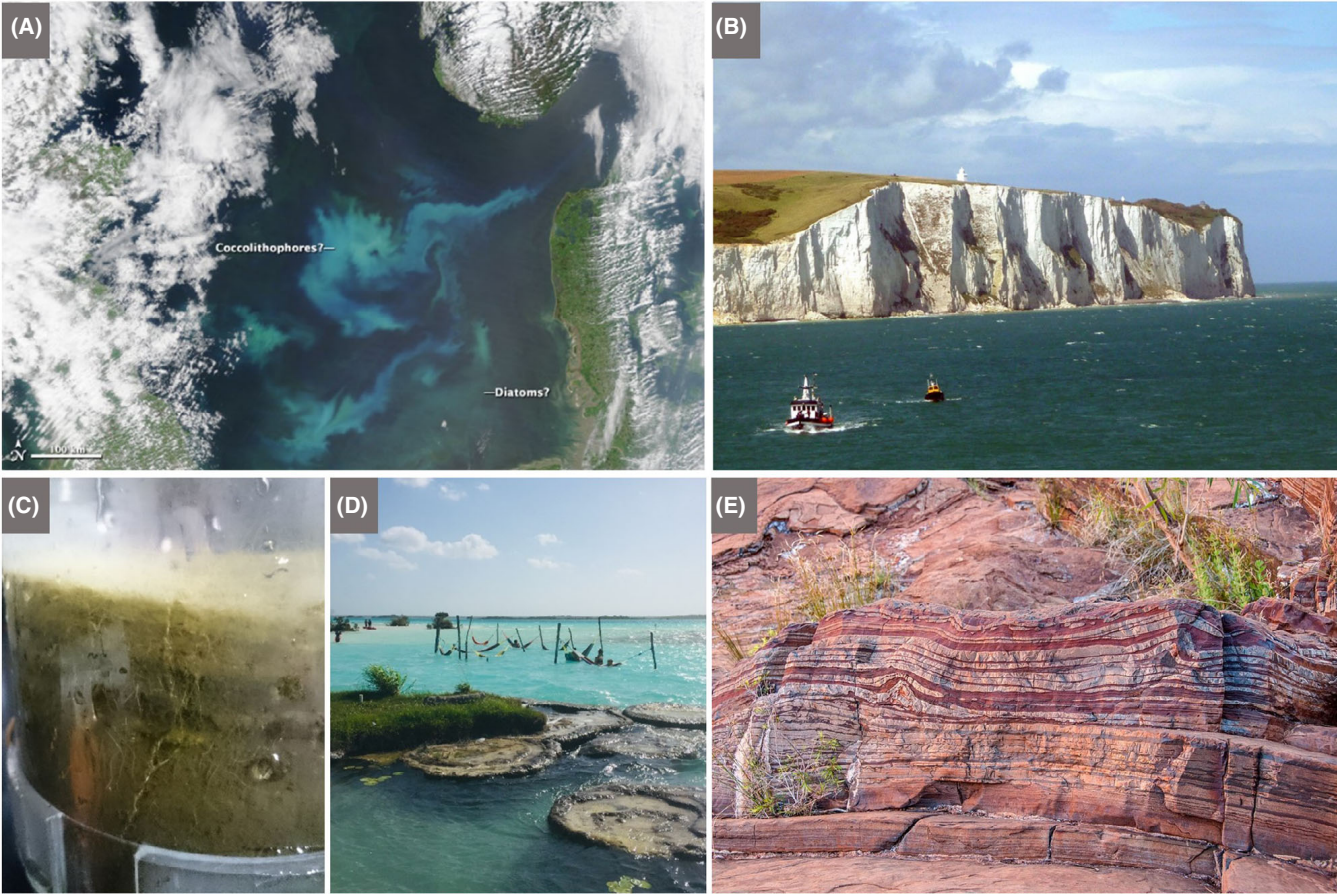
Blooming microbes A. Satellite image of a milky white bloom of the phototropic coccolithophore, *Emiliania huxleyi*, in the North Sea. This eukaryotic microalgal species has a calcium carbonate wall which, when the bloom collapses (due to death caused by virus attack and/or grazing), can sink to the seafloor. Thus, coccolithophores play a role in sequestering carbon from the atmosphere and transferring it to marine sediments. The greener bloom at the bottom of the image may be dominated by diatoms, which, owing to their glassy silica walls, can also be manifest in the rock record as diatomaceous earth. B. The White Cliffs of Dover, UK, made of chalk from the calcium carbonate walls of coccolithophores and foraminifera that settled to the sea floor in the Cretaceous period C. The giant bacterium, *Thiomargarita namibiensis,* can be seen in this sediment core from the Benguela Upwelling System of the coast of Namibia. It appears like white ‘strings of pearls’, i.e. a string of connected cells, which can be seen in the sediment. Individual cells are ~ 0.4 mm in diameter, and so, unusually for a bacterium, can be seen with the naked eye. It is non‐motile, and so relies on currents to deliver its terminal electron acceptor, nitrate, which it accumulates in a large vacuole. The sulfide, which provides its energy source, is abundant in the deeper anaerobic zone due to its production by sulfate‐reducing bacteria. There is also a thick, jelly‐like biofilm at the top of the sediment. Image width is ~ 5 cm. D. Fossilization in action. Giant microbialites that have formed over the last several thousand years due to microbial activity coupled with high carbonate concentrations in freshwater Laguna Bacalar in the Yucatán Peninsula of Mexico. Cyanobacteria are the dominant photosynthetic microbes, whose activity raises the local pH, which, together with activities of the associated heterotrophic microbial community, encourages carbonate precipitation. The sticky extracellular polymeric substances produced by the microbes also trap detrital grains. Microbialites can have accretionary layers (stromatolites) or a more clotted structure (thrombolites), and both are abundant in the rock record, with some dating back to ~ 3500 million years (predating the evolution of oxygenic photosynthesis, and thus raising questions about the microbes responsible for their formation). The layers or clots typically represent cyanobacterial biofilms or colonies respectively (Gischler *et al.*, 2008). E. Banded Iron Formation at the Fortescue Falls, Australia, with stripes rich in iron (darker bands) and silica (lighter bands) formed from oxidized iron sinking to the seafloor, mainly in the Archaean Eon. The way in which the ferrous iron minerals were oxidized in the early Earth is still under debate. The mechanism may have been oxygenic photosynthesis by the ancestors of modern cyanobacteria or iron‐dependent photosynthesis, which neither requires nor produces oxygen (Robbins *et al.*, 2019; Thompson *et al.*, 2019). *Photograph A by NASA (* https://earthobservatory.nasa.gov *), B by Immanuel Giel under the Creative Commons Attribution‐ShareAlike 3.0 Unported licence, C by Natalie Hicks, D by Etienne Low‐Décarie and E by Graeme Churchard under the Creative Commons Attribution 2.0 Generic licence.*

Fossilized calcium carbonate shells of eukaryotic microbes (especially coccolithophores and foraminifera) are the main component of chalk, which serves as a building material (e.g. parts of Westminster Abbey in London) and is still used for writing on blackboards in many classrooms across the world. Children are fascinated when they realize that such everyday objects, the rock beneath their feet and iconic natural formations, such as the White Cliffs of Dover (Fig. 10), are the remnants of microbes that bloomed in the oceans. The rock dolostone is a widely used building material, with examples including York Minster (UK) and the Western Wall in Jerusalem. Unlike chalk, the shells derive from larger, multicellular organisms, but microbes, specifically anaerobic sulfate‐reducing bacteria in marine sediments, are understood to be responsible for much of the formation of dolostone’s main mineral, dolomite (CaMg(CO_3_)_2_) (Krause *et al.*, 2012). Many other remnants or signs of microbial activity appear in the rock record, such as microbialites and banded iron formations (Fig. 10).

**Fig. 11:**
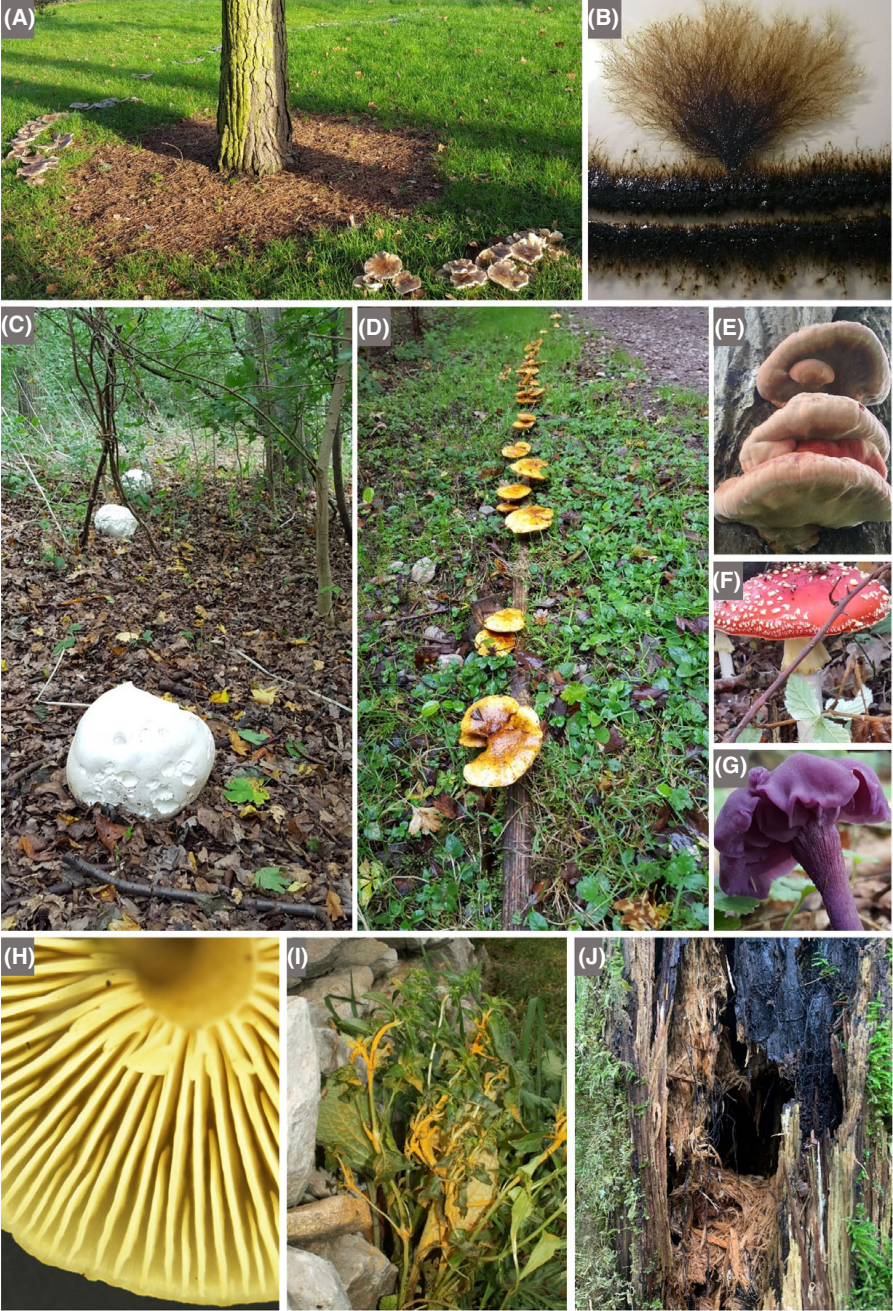
Fungi A. ‘Fairy ring’ of an unidentified Basidiomycete fungus around a pine tree at the University of Warwick, UK, a surface manifestation of a mycelial network belowground. B. Colony of the halophilic, melanin‐rich black yeast/fungus *Hortaea werneckii* (field of view is ~ 1 cm^2^). C. Three giant puffball fruiting bodies (*Calvatia gigantea*) in Wivenhoe Woods, UK, each ~ 30 cm in diameter. The hymenium, i.e. the tissue layer from which spores form, is internal. A specimen of this size would release around five trillion spores (Li, 2011). D. Basidiomycete fruiting bodies on the rotting, wooden edging of a path. E. Basidiomycete fruiting bodies of the shaggy bracket (*Inonotus hispidus*)*,* which has its hymenium housed in pores, are seen here on the trunk of a tree in northern England. Hispidin, a phenolic metabolite produced by this species, is an antioxidant with potential pharmaceutical applications. F. Fly agaric (*Amanita muscaria*) fruiting body in Wivenhoe Woods, UK. G. Amethyst Deceiver (*Laccaria* sp.) fruiting body in Wivenhoe Woods, UK. H. Stereoscopic image of the underside of a Basidiomycete fruiting body with the hymenium housed in gills (*Hygrophorus* sp.; radius is ~ 1 cm). I. Rust fungus (most likely *Puccinia aristidae*) on *Chenopodium album* in a garden in St. Saturnin‐les‐Avignon, France. The copious urediospores have dusted the stones behind the plant, colouring them orange. J. Fungal rhizomorphs of the saprophytic and root parasitic honey fungus (*Armillaria mellea*) on a rotting tree trunk in Broc, Switzerland. These structures are very resistant and are able to explore their environments on metre scales to scavenge for nutrients and then to translocate fluids bidirectionally. There are many excellent mycology teaching resources, notably ‘Mushrooms primary school activity pack’. Photographs *A, C, D, E, F and G by Terry McGenity, B by Polona Zalar, H by Saskia Bindschedler, I by Cindy Morris and J by Andrea Lohberger.*

**Fig. 12:**
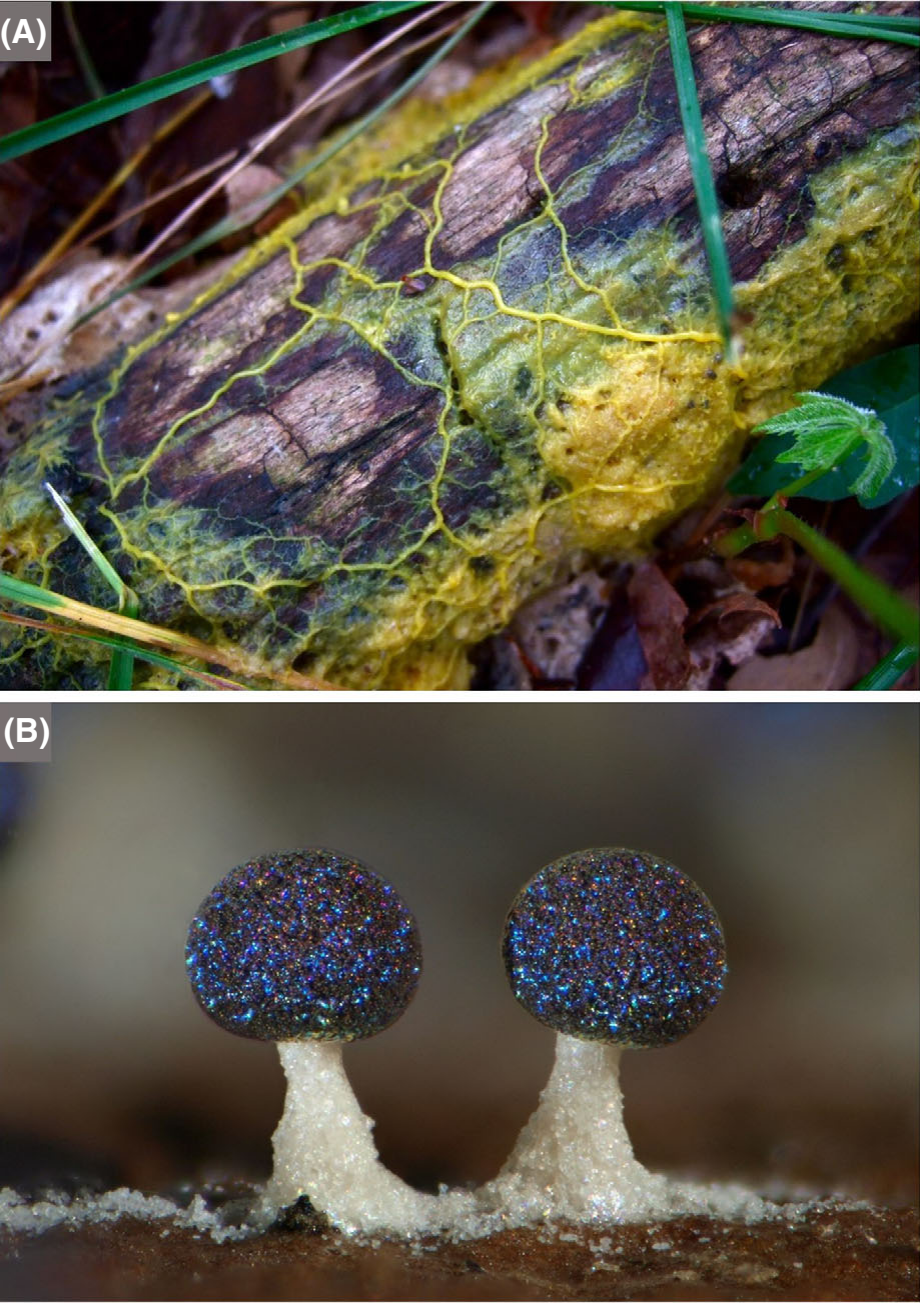
Slime moulds A. *Physarum polycephalum* plasmodium (single‐celled multinucleate amoeba capable of rapid streaming) on rotting wood. B. Fruiting body (sporocarp) of *Diachea radiata* in leaf litter of the evergreen tree, *Bellucia grossularioides,* in regrowth forest near Yangambi in the Democratic Republic of the Congo. Excellent videos of these fascinating organisms can be seen on the internet. *Photograph A by Frankenstoen, under the Creative Commons Attribution 2.5 Generic licence, and B by Myriam de Haan, Meise Botanic Garden.*

Two diverse and distinct eukaryotic groups, fungi with their hyphal networks and fruiting bodies (Fig. 11), and slime moulds with their massive multinucleate cells and fruiting bodies (Fig. 12), provide a fascinating visual and often beautiful portrayal of the microbial world. Important information about the state of world’s fungi is provided by Willis (2018) and Kew Gardens – Protecting precious fungi web page, while Glime (2019) provides well‐illustrated information about slime moulds.

Fungi are attractive to children given that they are often morphologically distinctive, and the basidiomycete fruiting bodies are steeped in folklore; for example the insecticidal, hallucinogenic, home for elves, *Amanita muscaria* (fly agaric) and fairy rings formed by numerous fungal species. Fruiting bodies are also fascinating because they are dynamic, changing form and often colour and odour as they age. They can sometimes be seen releasing vast numbers of spores (e.g. puffballs). Fungal hyphal networks can be seen with the naked eye by looking at mouldy bread, degrading wood and beneath forest litter. Likewise, fungal rhizomorphs, such as those from *Armillaria mellea* (honey fungus), can be identified in the field. The beauty of ephemeral fruiting bodies, along with the extent and longevity of many fungal mycelia (the largest organisms are fungi: the honey fungus mycelium can extend over several kilometres), fascinate children and can stimulate discussion about fungal life cycles, chemical defence and signalling (and link to production of antimicrobials), positive and negative interactions with plants and roles in biodegradation.

#### Suggestions

Microbes are all around us, and local universities, wildlife organizations, amateur enthusiasts and field centres/councils are frequently eager to offer advice and tours/activities. Here, we list a range of environments where microbiology can be seen in action.

**Fig. 13:**
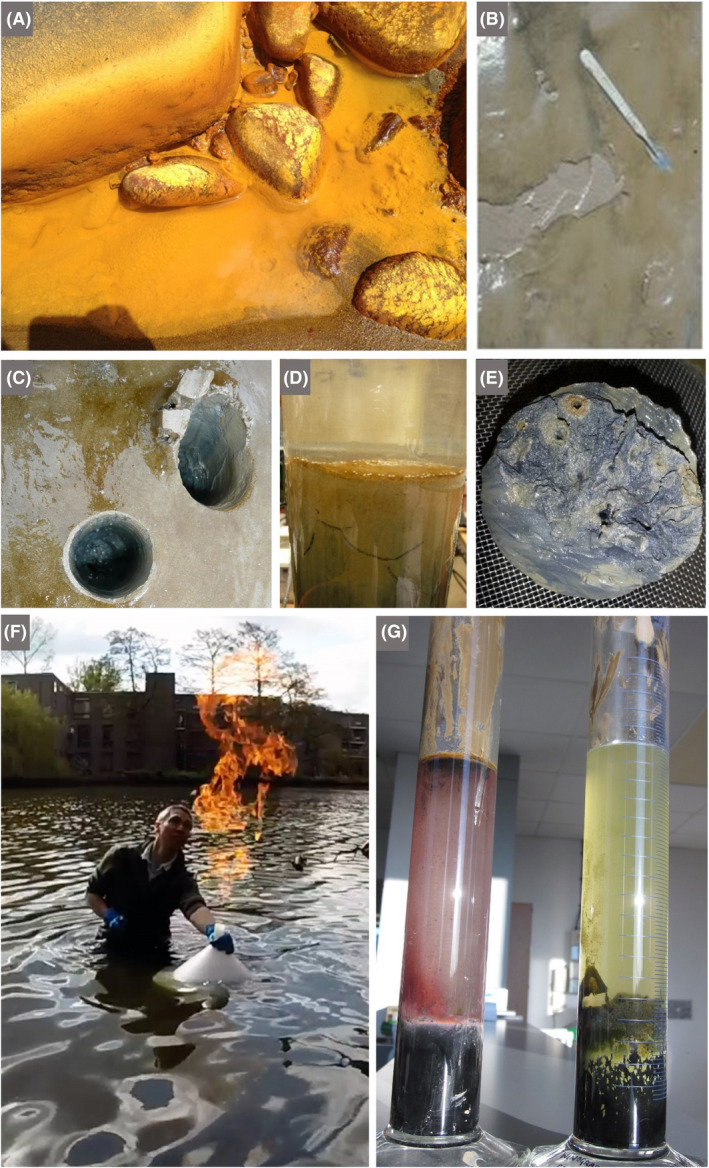
Microbial manifestations of their phototrophic and reduction–oxidation activities A. Boulder and pebbles in Río Sucio (Dirty River) in Costa Rica are coloured orange due to the presence of schwertmannite, a mineral generated by the activity of iron‐oxidizing bacteria, such a *Gallionella* spp., which use reduced iron as a form of energy, oxidizing it to form rusty minerals (Arce‐Rodríguez *et al.*, 2017). B. Peel of mudflat sediment showing a thick gelatinous biofilm rich in diatoms (silica‐walled eukaryotic phototrophic microalgae) on top of the clay‐rich sediment. Diatoms are the main primary producers in this ecosystem and, by the production of sticky extracellular polymeric substances, they stabilize the sediment, thus limiting coastal erosion. C. Cored sediment from Tillingham mudflat, UK (holes are ~ 8 cm diameter), showing the golden mats formed by diatoms on the surface, and the transition to grey‐black sediment at depth caused by anaerobic sulfate‐reducing bacteria releasing sulfide that reacts with iron to form black iron sulfide. D. Extracted sediment core from C, incubated in the light, showing how the diatoms and other phototrophic microbes produce bubbles of oxygen visible at the surface. E. A transverse slice of the sediment shown in D, illustrating how burrowing animals such as the ragworm (*Hediste diversicolor*) can introduce oxygen deeper into the sediment and thus encourage the activity of aerobic microbes, such as iron‐oxidizing microbes, leading to a ring of rust around the holes. F. Burning methane from the lake at the University of York, UK. The sediment was stirred to release methane bubbles (produced by anaerobic methanogenic Archaea), which were collected in a funnel and set alight. This phenomenon can occur without human assistance, with the resultant ghostly light engendering myths and legends worldwide, such as Will‐o'‐the‐Wisp and *ignis fatuus*. G. Winogradsky columns set up with cellulose and sediment from an Essex salt marsh. The left‐hand column is dominated by purple sulfur anoxygenic phototrophs, while the right‐hand column is dominated by green sulfur anoxygenic phototrophs, both of which are Bacteria that use light as an energy source, but instead of splitting water and generating oxygen (as in the diatoms in D) they split hydrogen sulfide. The sulfide is produced by anaerobic sulfate‐reducing bacteria found in the black sediment. *Photograph A by Max Chavarría, B by Graham J.C. Underwood, C, D, E and G by Terry McGenity and F by Paul Shields and James Chong.*

- Old buildings, monuments, walls, etc., with growths of algae, fungi, Bacteria, Archaea and lichens (Fig. 9)
- Local cemeteries can be visited to introduce lichens, including a discussion on the nature of their symbiosis, their use as indicators of air pollution and how their colony size and diversity may relate to the date when the headstone was erected (Samsudin *et al.*, 2012).
- Degrading and discoloured paintings and tapestries in old buildings
- The built environment – fungal spores, mould in showers or around school sinks, decay, dry rot and food spoilage
- Geological formations (potentially) resulting from microbes and their activities – e.g. chalk, stromatolites, diatomaceous earth and banded iron formations (Fig. 10) (ask whether microbes built St. Paul’s Cathedral in London to start a conversation about the role of microbial biofilms in making oolitic sand grains which lithify to form oolitic limestone)
- Modern‐day stromatolites (ask why they are not as common now as in the past) (Fig. 10)
- Soils raise numerous microbiological questions: what is the microbial role in soil and ped formation, structure and health; which microbes and their volatile compounds are responsible for the smell of soil; which microbes form desert soil crusts (Fig. 9); and what other macroscopic microbial marks can be seen (e.g. nitrogen‐fixing root nodules; Fig. 5)
- Forest/grassland excursions to see and discuss fungi and slime moulds: fungal identification, fruiting bodies and their spores (making spore prints); plant‐mutualistic fungi with ectomycorrhizal structures; pathogenic fungi with spots on leaves (powdery mildew, maple tar spot, *Armillaria* rhizomorphs); saprophytic fungi (networks under leaf litter, white and brown rots); and slime moulds as evolutionarily distinct from fungi but often growing in similar habitats feeding on microbes (Figs. 11 and 12)
- Salt marshes, mangroves, mudflats and their microbial mats (distinguish diatoms, euglenoids, etc.; ask whether there are any anoxygenic bacterial phototrophs and use material to make a Winogradsky column; and look for white mats of sulfur‐oxidizing bacteria) (Fig. 13)
- The coast, river and soil, e.g. identifying and quantifying plastics, and asking why polymers (bio)degrade at different rates
- Eutrophic/non‐eutrophic water bodies (distinguish different eukaryotic algae and cyanobacteria)
- Anaerobic ponds versus aerated streams with constructed weirs (note the smell and bubbles from the sediment)
- Coral reefs (photosynthetic *Symbiodinium* endosymbionts; microbe‐rich mucous layer)
- Bioluminescent fungi and bioluminescence at sea caused by free‐living eukaryotic dinoflagellates or bacterial endosymbionts of squid and fish (follow this with isolating bioluminescent bacteria, e.g. by swabbing from squid eyes onto marine agar plates)
- Caves with biofilms, biominerals (e.g. moon milk), snottites, paintings made with microbial pigments and biodeteriorating cave art
- Compost heaps (ask why are they hot)
- Insect nests, e.g. anthills, termite mounds or beehives to discuss: insect–microorganism associations, fungal gardens as a food source, microbiological control of pathogens for the insect colony, and sources of novel antimicrobial drugs
- Sulfur springs (anoxygenic phototrophic bacteria, filamentous sulfur‐oxidizing bacteria, sulfate‐reducing bacteria and the smell of rotten eggs)
- Iron springs (iron oxidation and microbial sources for reduced iron)
- Iridescent sheens on the surface of ponds, puddles and ditches that are sometimes confused for oil spills, but are caused by iron‐oxidizing bacteria and lipids released from cells
- Degraded industrial landscapes, e.g. rivers acidified by mine drainage (e.g. Rio Tinto, Spain), former mine sites, gasworks and chemical plants undergoing restoration by bioremediation or phytoremediation
- Extreme environments, especially where dominant microbial communities are visible, e.g. red salterns, salt lakes (Fig. 2), alkaline lakes (with flamingos feeding on the frequently dominant cyanobacterial species, *Arthrospira platensis*), slimy and colourful thermal springs, and pink or red snow, also called watermelon or blood snow (often with the dominant microalgal species *Chlamydomonas nivalis*, which is in the same family as *Dunaliella salina*; Fig. 2)

**Fig. 14:**
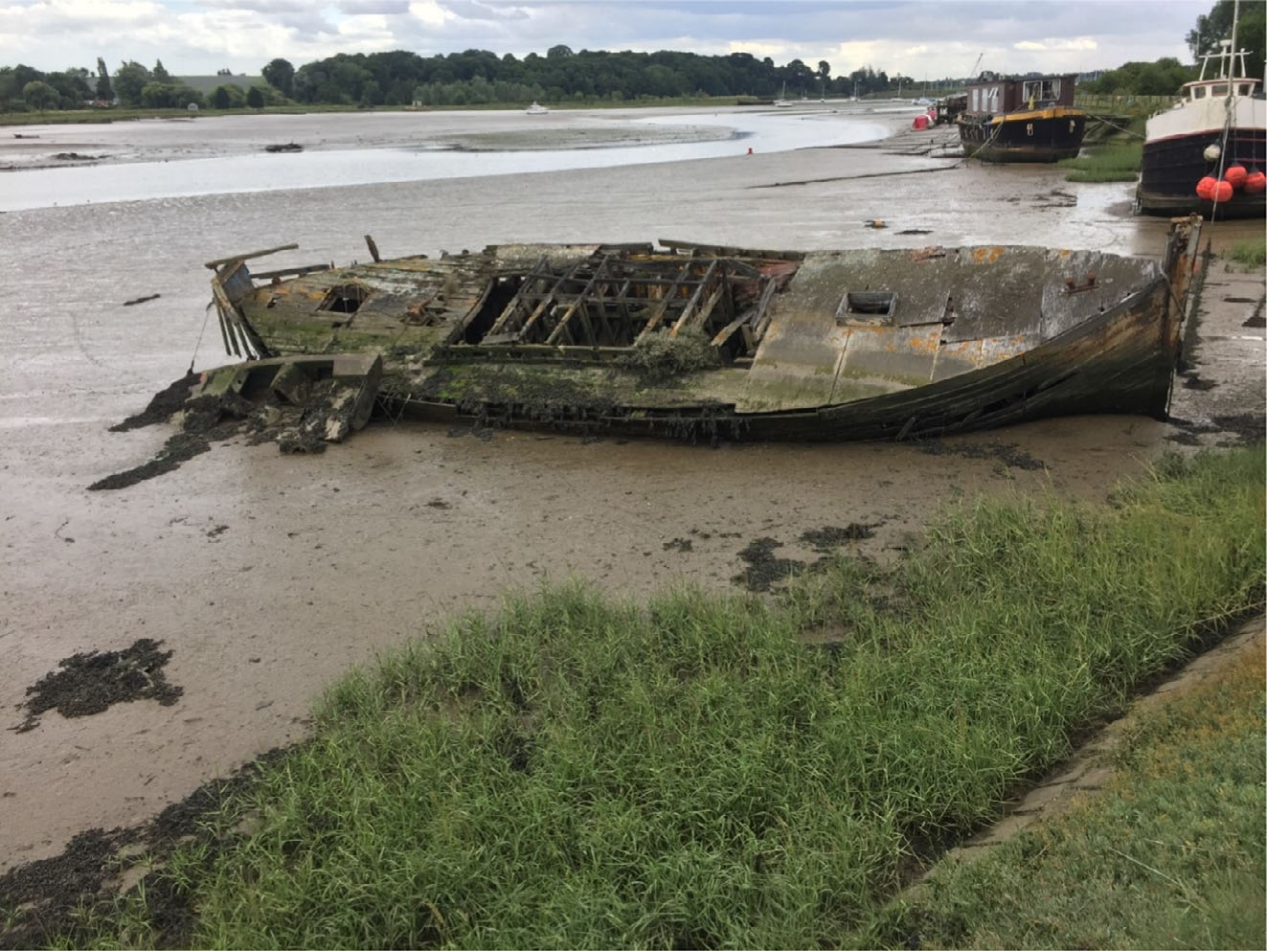
Decaying objects can be found in all sorts of environments and provide the basis for discussing the underlying microbiology of decay and nutrient recycling. For example, children can be asked who is eating this rotting boat on the bank of the tidal River Deben, near Woodbridge, UK, and how they do it, thereby introducing children to extracellular and intracellular enzymes. Children can be encouraged to consider where else they encounter rotting and recycling (e.g. leaves in the forest, food in the fridge, fences in the garden, waste in landfill sites), when it is beneficial/damaging and how it can be encouraged/prevented. *Photograph by Kenneth Timmis*

#### Some discussion topics

While exploring the built environment with children, it is pertinent to ask them: why wooden structures decay more rapidly than those made of stone, why they need more protection from microbes, and why wooden window frames and fascias have largely been replaced with plastic. The role of cellulolytic and ligninolytic microbes in deterioration should naturally follow from these questions (Fig. 14). Thereafter, the plastic problem is likely to be a topic of discussion, with questions such as: why are most plastics resistant to biodegradation, what are the global problems caused by non‐/slowly biodegradable plastics, from where can we get biodegradable plastics (microbes, plants, crustaceans, etc.)? These questions may provoke students to design experiments to test the biodegradability of different polymers (Fig. 8) and thereby lead them to establish cultures to grow polymer‐degrading microbes.

If age‐appropriate, the eternal cycle of life and death, and the involvement of microbes in it, can be discussed during a history trip to a local cemetery. This may usefully lead into a discussion of cremation, carbon footprints of different means of disposing of our earthly remains (including composting), the issue of mercury fillings of teeth and the deposition of mercury downwind of crematoria, mercury resistance in microbes and the application of mercury‐resistant microbes in the detoxification of mercury‐polluted waste streams (von Canstein *et al.*, 1999).

It is valuable to combine different field activities to obtain a more complete and integrated understanding of a system’s ecology, providing opportunities to consider food webs, nutrient cycling, etc. (Barberán *et al.*, 2016). When investigating a rock pool, in addition to using a magnifier to view zooplankton, a microscope can make microbes visible and initiate a discussion about their abundance and importance in diverse ecosystem processes, pointing out that every cubic millimetre (the size of a poppy seed) houses approximately 10,000 viruses (Suttle, 2007), 600 Bacteria (including 150 cyanobacteria), nine microalgae, six Archaea (Karner *et al.*, 2001) and on average less than one protozoan (Zubkov *et al.*, 2007). Then, if you were able to sample 100 m below the seawater surface, the relative contribution of Archaea would increase greatly, in some locations occurring in the same abundance as Bacteria (Karner *et al.*, 2001).

**Fig. 15:**
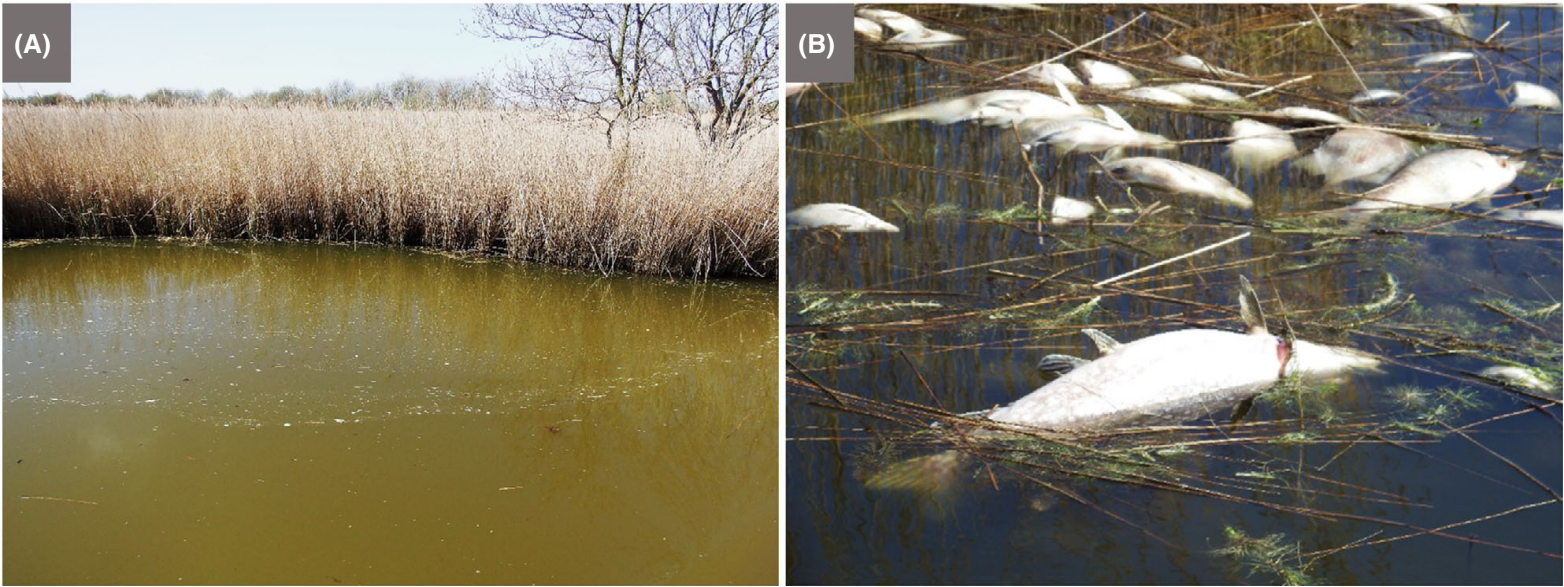
Algal blooms and harmful algae A. Bloom of the dinoflagellate, *Prymnesium parvum* on the Norfolk Broads, UK. This dinoflagellate is a photosynthetic eukaryotic microalga that produces a toxin, which can lead to significant economic damage. B. A few of the ~ 30,000 fish killed by the blooming dinoflagellate, *Prymnesium parvum* on the Norfolk Broads, UK, in April 2015. *Photographs by Martin Rejzek.*

Freshwater lakes are generally transparent due to relatively low concentrations of the macronutrients, nitrogen and phosphorous, limiting the growth and abundance of phototrophic microbes. But, with a seasonal, or sporadic, input of nutrients (e.g. run‐off from agricultural land), the water body may turn a deep, turbid green colour owing to dense blooms of phototrophic microbes, such as cyanobacteria or eukaryotic microalgae, a process known as eutrophication (Chislock *et al.*, 2013). As microalgae die, aerobic heterotrophic microbes consume their organic remains leading to the depletion of oxygen, which may result in the death of oxygen‐dependent aquatic animals, including fish. Moreover, some microalgae produce toxins that poison animals that live in the lake (Fig. 15) and, when this happens, children are prohibited from swimming or fishing in the lake. Having the flexibility for the class to go out and inspect such a bloom, especially when it is making the local news, can provide multiple educational benefits. Not only will such an excursion stimulate a discussion about the underlying microbiological processes, it should also lead to an exploration of the link between our food requirements, farming practices and their environmental effects. Moreover, interested families are likely to question their children/siblings about their visit, and thus continue the learning process for the student and their family.

### Museums, zoos, aquariums and botanical gardens

#### Context

**Fig. 16:**
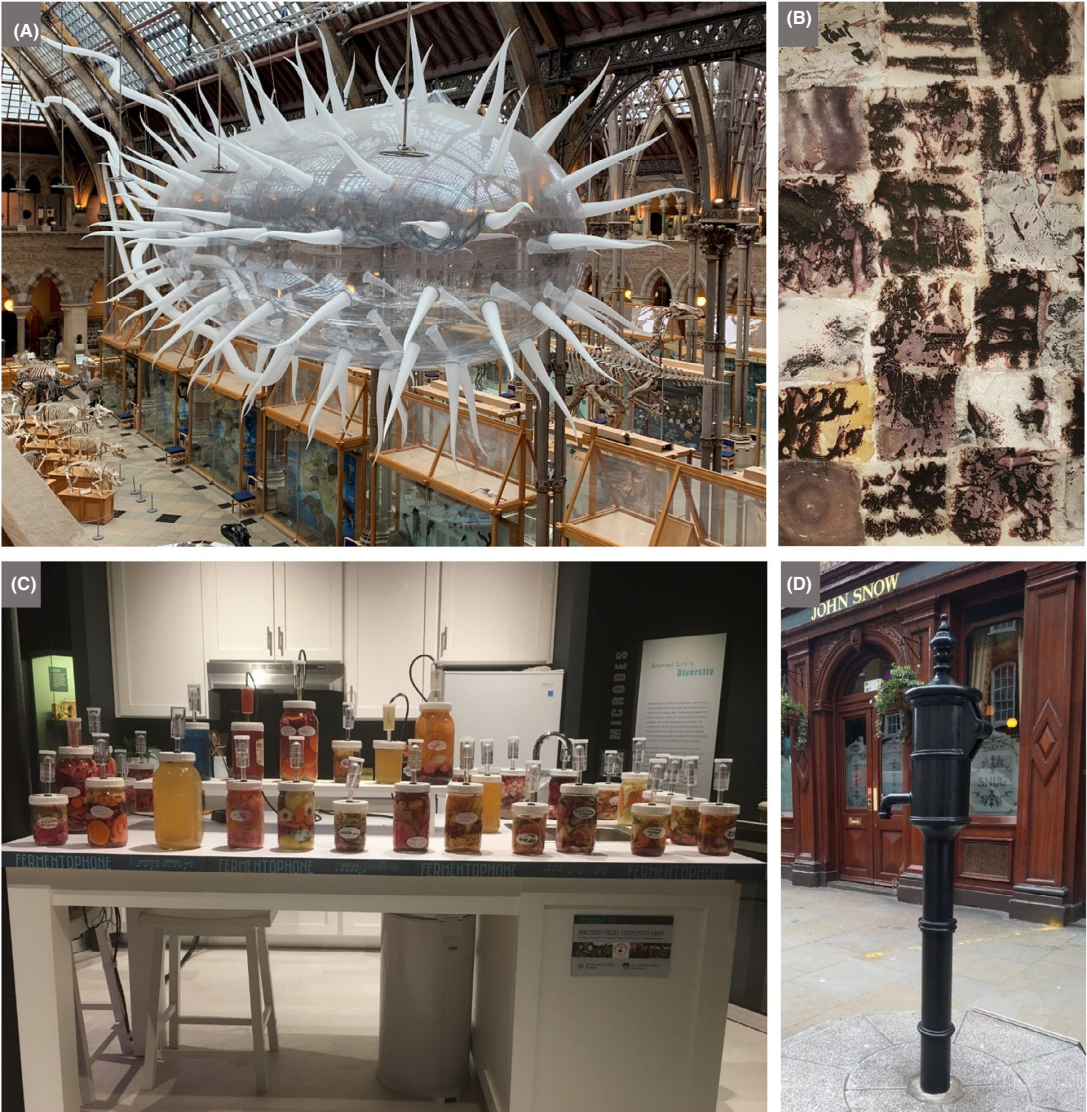
Microbial exhibits A. The giant *Escherichia coli* sculpture created by Luke Jerram was the centrepiece of the Bacterial World Exhibition. The 28‐m‐long piece is 5 million times bigger than a real microbial cell and highlights some of its external structures such as flagella and pili. B. ‘The Hunt for New Antimicrobials’ created by Anna Dumitriu in collaboration with Maggie Smith and Nicholas Read was part of the BioArt and Bacteria exhibition. This framed piece is composed of silk, sterilized wild‐type and genetically modified *Streptomyces* bacteria, wood, card and glass. Both exhibits shown in A and B were on display at the Oxford Museum of Natural History, UK. Redfern *et al. *(2020) provide more examples of exhibits that aim to highlight the issue of antibiotic resistance. C. The Fermentophone, designed by Joshua Rosenstock, consists of jars of fermenting fruits and vegetables producing CO_2_ bubbles, which are picked up by submerged microphones and converted to different sounds, creating a relaxing and aesthetically pleasing, as well as edible, display. Moreover, visitors can make their own ferments and add them to the living exhibit, shown here in action at Harvard Museum of Natural History, USA. D. The Broad Street Pump replica outside the John Snow Public House in London, UK, is an example of microbiology history – where the field of epidemiology was founded. By meticulous detective work, the Yorkshireman, John Snow, identified the pump as the source of a cholera outbreak in 1854, saving lives in the short term by the removal of the pump’s handle and in the long term by recognizing the role of sewage as the source of the waterborne disease. There are famous microbiologists and microbiological stories associated with many towns, which teachers can include in an excursion to enliven the subject. *Photograph A by André Antunes, B by Carol Verheecke‐Vaessen, C by Joshua Rosenstock and D by Mairi McGenity.*

A core role of most museums is to support school visits, though in many cases they have the ‘visible’, rather than the ‘invisible’, on show. However, those with artefacts, ancient or recent, are almost always confronted with issues of conservation, including the mitigation of microbe‐mediated deterioration. Thus, a visit to such museums may be combined with exposure to microbially based conservation programmes. Moreover, there are museums and exhibitions specifically dedicated to microbes and their activities, reflecting the public’s fascination with microbes, and museums’ remit to promote and contribute to Sustainable Development Goals (McGhie, 2019; Fig. 16). Then, there are zoos, aquariums and botanical gardens, some of which may have expertise in microbial infections, biological control methods and microbiomes; fungi are sometimes showcased in botanical gardens (e.g. Kew Gardens in London; Willis, 2018), while slime moulds have recently been introduced into the collection of the Parc Zoologique de Paris. Visits to livery stables, animal sanctuaries or dog/cat homes may provide a more local and cost‐effective means of combining children’s typical love of animals and learning about the microbial contribution to health and disease. However, for such excursions the expertise in microbiology will most probably have to come from the teacher or an outside recruit.

#### Suggestions

The school liaison officer in local museums, zoos, aquariums or botanical gardens should be able to advise on microbiological research, exhibits or activities that could form part of a school visit. Here, we highlight some microbiology‐focused museums and exhibitions. As the home of so many microbiological innovators, and some of the more unusual museums, it is unsurprising that the Netherlands hosts the world’s biggest museum dedicated to microbiology; the Micropia Museum in Amsterdam is a rarity among museums in focusing on microbiology in such an engaging and impactful way. Another can be found at the Instituto Butantan in Brazil. The Wellcome Trust Collection, London, UK, has for many years revealed to the public the role of microbes in human disease and health, and there is growing adoption of itinerant microbiological exhibits and activities in other museums, such as the BioArt and Bacteria exhibition, and the Bacterial World exhibition with its giant inflatable *Escherichia coli* (Fig. 16), the Microbiota exhibition at La Cité des Sciences in Paris, France, the Outbreak exhibition at the Smithsonian Museum of Science and the World in a Drop: photographic explorations of microbial life in Harvard Museum of Natural History, USA. The Eden Project (Cornwall, UK) has a focus on Invisible Worlds. The Bioluminescent Photograph Booth was a touring interactive exhibition using bacterial bioluminescence to enable ghostly images of the human subjects. An innovative, immersive exhibit at the Exploratorium Science Museum in San Francisco, USA, used the light‐sensitive behaviour of a *Euglena* species to give the impression that the eukaryotic microbe was dancing with visitors while in reality avoiding their shadow (Lam *et al.*, 2019). More unusually, microbial metagenomics data sets have been set to music (Larsen, 2016), and bubbles from fermentative microbes have been used to play relaxing tunes in an interactive exhibit called a Fermentophone (Fig. 16). Importantly, there are also museums associated with inspirational microbiologists, e.g. Beijerinck Museum in Delft (the Netherlands), Museum at the Robert Koch Institute in Berlin (Germany), Alexander Fleming’s Museum in London (UK) and Louis Pasteur’s house in Arbois (France).

#### Some discussion topics

**Fig. 17:**
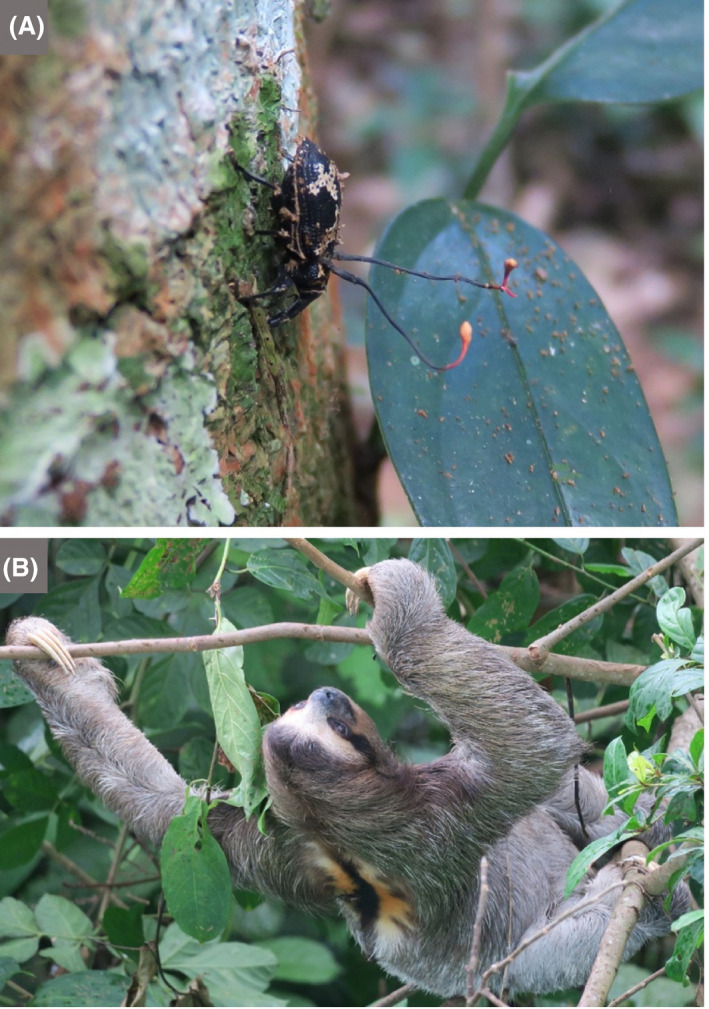
Unusual and visible animal‐microbial interactions A. An adult weevil on a lichen‐covered tree (in Alta Floresta, Mato Grosso State in the southern section of the Brazilian Amazon) infected with the fungus *Ophiocordyceps curculionum.* The objects that look like antennae with orange/red ends are actually fungal fruiting bodies. *Ophiocordyceps* species are parasites of beetles and other insects. The behaviour of the infected beetle is changed to make it land at a certain height (~1 m) on a tree trunk. When infecting ants they modify their host’s behaviour, turning them into ‘zombies’ that move along the vegetation, locking their mandibles into precise locations on the underside of leaves where fungal development is optimized. The infected, fixed ants are generally positioned above foraging trails so that spores from *Ophiocordyceps* are likely to infect other ants (Araújo and Hughes, 2019). B. A male brown‐throated three‐toed sloth with green fur. Sloths’ hair has unique transverse cracks that retain moisture and thus support the hydroponic growth of a diverse range of eukaryotic microalgae and cyanobacteria. The highly digestible and lipid‐rich microalgae are eaten by sloths, supplementing their poor diet which otherwise consists exclusively of leaves (Pauli *et al.*, 2014). *Photographs by Margaret Adams.*

Children tend to be aware of the value of higher organisms, such as honey bees as pollinators, and recognize the power and beauty of endangered sentinel species like Amur leopards. Older children also understand the wider value of biodiversity and its conservation. Thus, a visit to a zoo, aquarium, botanical garden or a museum provides an ideal opportunity to encourage debate about the contribution of microbes to the health and conservation efforts of animals (Bahrndorff *et al.*, 2016; Trevelline *et al.*, 2019) and plants (Berg and Raaijmakers, 2018). The difficulty in keeping some animals in zoos, such as three‐toed sloths, has been attributed to obtaining the right diet to support their specialized internal and external microbiomes (Dyer, 2003; Pauli *et al.*, 2014; Dill‐McFarland *et al.*, 2016; Fig. 17). Three‐toed sloths are fascinating from another microbiological standpoint: they carry around in cracks in their fur portable microbial ‘vegetable gardens’ which provide valuable nutrients on demand (Fig. 17). Flamingos in zoos may lose their pink pigmentation, as they are not feeding on their usual carotenoid‐rich cyanobacteria and microalgae (directly or indirectly), which impart the pink colouration in their natural habitat (Dyer, 2003).

Emblems for schools, towns, states, etc., can deliver powerful messages about issues of local importance and serve as sources of civic pride. New Jersey has antibiotic‐producing *Streptomyces griseus* as its state microbe, while Oregon, famous for its craft beer production, has *Saccharomyces cerevisiae*, so microbes have been adopted as important emblems. A microbiology excursion may thus be enhanced by asking children to think about which microbe deserves to be considered for their school, town or state emblem. Older children can be challenged to consider which microbe they would most want to conserve and why, e.g. because of its rarity, beauty or value to humans. This question is sure to evoke a response that can be explored further by discussing why we are innately less likely to want to conserve small organisms, yet why we should do so.

It will be informative to consider the consequences of microbial extinctions and how to conserve microbes; for example, by focusing on habitats, the habitats’ genetic resources or on cultured microbes (Cockell and Jones, 2009; Paul and Mormile, 2017). In the case of protecting or preserving habitats, which is of course key to protecting endangered species of plants and animals, children can/should be encouraged to develop a sense of pride in their local ecosystems as they start to learn about the microbes within. When considering preservation of microbial cultures, children can be introduced to the valuable roles played by Microbial Domain Biological Resource Centres (Antunes *et al.*, 2016), which can also make for an interesting excursion.

Finally, most zoos have ants, usually tropical leafcutter ants, which can elicit discussions about their interactions with microbes, notably the different ways that they farm fungi, and the role of associated antibiotic‐producing bacteria (Chomicki and Renner, 2017; Whitaker and Stolzmann, 2019). Children are particularly fascinated by interactions that induce weird behavioural changes in animals, for example the fungus *Ophiocordyceps* sp. that uses carpenter ants as a vehicle for reproduction and dispersal – creating so‐called zombie ants (Hughes *et al.*, 2011; Araújo and Hughes, 2019; Fig. 17). And, of course, this can trigger discussions about the varied behavioural effects that *Wolbachia* species have in diverse insect hosts (see Section 5).

## Tips for preparing an excursion

Useful advice on running and evaluating activities, including site visits and field trips, is given by Behrendt and Franklin (2014) and the Council for Learning Outside the Classroom (LOtC). There is a large body of evidence to show that active learning promotes student performance (Freeman *et al.*, 2014). Therefore, in order to get maximal benefit from excursions, it is important for the host to develop age‐appropriate and engaging activities. When teaching microbiology to young children, the focus should be on discovery, the wonder and amazement of microbes, nurturing respect for microbes and understanding that they are members of the natural world like all other more familiar organisms. Then, children can be introduced to the roles that microbes play and services that they render, such as recycling and degrading. For older children, there can be more focus on global issues and the networking/integration of microbial processes.

The following points provide some general, and some microbiology‐specific, tips that may be helpful when preparing for an excursion.

1. Inform yourself about the availability of excursion possibilities of microbiological interest within a practical radius of the school (excursions further afield may also be noted for special occasions or joint excursions with other schools).
2. Decide whether only one excursion or more can/will be made.
3. Familiarize yourself with the microbiologically relevant processes.
4. Contact multiple organizations and ask questions, such as:
  - whether an excursion is possible
  - which topics are to be covered
  - whether they have hosted school groups previously and, if so, who were the lead teachers. One or more of these should be contacted for an assessment and advice on how benefits might be maximized.
  - whether logistical issues exist, the size of rooms to be visited are adequate for the numbers in the group (a room with a capacity for eight is not useful for a class of 30, which will spill out into the corridor and not see/hear what is going on)
  - whether anything else of interest can be shown/discussed.
5. Decide which of the possibilities are the best options for the class, select the best and have at least one in reserve. Check out at least two, as indicated below, to obtain a comparison.
6. Make an appointment to meet the person(s) hosting/leading the on‐site visit. Ask if it would be possible to also meet any replacement staff who would take over, just in case illness or anything else prevents the person being available on the day.
7. During the visit, try to see first hand what will be shown and explained to, or experienced by, the class, and determine:
  - how they prepare a class visit
  - what specifically will be introduced and explained
  - whether the age and knowledge level of the children is appropriate for the visit, and whether the information to be presented during the excursion matches the requirements of the group
  - what opportunities exist for hands‐on activities
  - the proposed presenter:student ratios – try to have small groups to maximize interactions
  - what safety issues need to be considered in order to complete a thorough risk assessment
  - whether the facilities and necessary adjustments for any students with disabilities or special needs will/can be available.
8. Stress the value of: humour, interactivity and enthusiasm in presentations/explanations, hands‐on activities and allowing sufficient time for questions, discussions and moving around the site
9. Assess the ability of the presenter(s) to engage the children, communicate and create excitement and enthusiasm for the topic: outgoing, humorous (boisterous) types – entertainers – are to be preferred over quiet, academic types, and those unable to throw their voices to the back of the group.
10. Ask for possible excursion dates compatible with your own class schedule.
11. After checking more than one option, discuss the potential visits with colleagues who have experience of class excursions and/or microbiological expertise.
12. Select the one that will be the most informative, attractive and entertaining for the class, then fix the date and time of the visit. Confirm details of the topics to be presented to the class and check that replacement staff will be on stand‐by in case the responsible person(s) is(are) unavailable.
13. In a follow‐up discussion with the selected host:
  - explore any additional or alternative topics and approaches triggered by the visit that may be valuable for the visiting children
  - to prepare for the excursion, request literature, ideally including a lay summary, and explore online videos that deal with the microbial processes to be shown (several may be made available as part of the Microbiology Literacy Initiative).
14. Before the excursion, as far as possible teach the relevant microbiology to the class, discuss the visit and ask the children to prepare/suggest questions.
15. Design worksheets or obtain material from your host to be used during the visit, and include the opportunity to draw images under the microscope if relevant. Then explore follow‐up investigations after the visit.
16. Consider allowing the children to take home a microbiological memento, e.g. by making postcards or calendars from images of their agar cultures or micro‐/stereo‐scopic images.
17. Enjoy!
18. Afterwards, assess what went well and what could be improved for a future excursion.

## Importing excursions into school and virtual excursions

While the novelty of excursions provides unparalleled learning benefits, they are clearly associated with a logistical and financial cost. Where the logistics are inhibitory, or funds are lacking for this purpose, alternatives are available. The most obvious strategy is to invite into the classroom a speaker working in a microbiological area. A former student, especially if charismatic, who can easily relate to the class and local circumstances, can be particularly valuable. Such external speakers should use pictures, videos, props and, where possible, replace/intercalate a talk with practical activities, as appropriate for the age group; for example, Couto (2017) provides an imaginative way to introduce biofilms to 4‐ to 6‐year‐old children, and Vrentas *et al. *(2011) take students on an imagined MicroSafari. Such hands‐on and interactive activities (and more, e.g. an insect’s head with *Ophiocordyceps* growing out of it (Fig. 17), smelly food (Fig. 4), settling activated sludge (Fig. 6) and making electricity from microbes and mud) are particularly memorable and can evoke long‐lasting interest in microbiology.

Speakers and teachers can use a plethora of resources, especially via the internet (e.g. Guarner and Niño, 2016), to draw students into the world of microbes. Some videos illustrate important concepts that would be much more difficult to convey by other means. For example, large‐scale experimental design and the clever use of time‐lapse imaging resulted in a very powerful demonstration of bacterial evolution in action by use of the Microbial Evolution and Growth Arena (MEGA) plate (Baym *et al.*, 2016). National and international microbiological societies, together with many academic centres, provide excellent resources on their websites, and, moreover, given the importance of microbes to all scientific disciplines, other learned societies and organizations, from astrobiology to zoology, have microbiology‐focused resources. For example, the International Ocean Discovery Program (IODP) has useful resources for educating children about the deep biosphere. Public Health England’s e‐Bug initiative provides excellent material for school children of different ages, including lesson plans on antimicrobial resistance, the value of which in improving understanding of the issue by children in India has been shown (Fernandes *et al.*, 2019).

Social media, together with other media outlets, feature microbiology news stories that can precipitate class debates on topical and/or controversial issues. Social media also provides a vehicle for teaching microbiology, using a range of approaches from informative cartoons to interactive courses (López‐Goñi *et al.*, 2016). Comics, with their visuals, narrative and metaphors, are an enjoyable way to learn about microbiology (Scavone *et al.*, 2019). There is a growing availability of games, such as *Top Trump*‐style games, like Comic Bacteria’s Micro‐Match and the Eden Project’s Invisible Worlds, as well as online games, such as those developed by Public Health England’s e‐Bug initiative (Farrell *et al.*, 2011), the Centre for Disease Control (Solve the Outbreak) and the American Museum of Natural History (Bacteria in the Cafeteria). Foldit is a citizen‐science game (aimed at older children and adults) that takes advantage of human pattern recognition and puzzle‐solving skills to determine protein structures (Cooper *et al.*, 2010). A current challenge is to design protein structures that would block the coronavirus spike protein’s interaction with host cells, as a step towards antiviral drug development. Reference to the use of microbiology in science fiction or popular culture can spark the interest of children, but should generally be considered as a starting point for discussion, e.g. what are the microbes in the pink salt lakes that are the subject of a ‘Go Jetters’ episode? However, the improbability of humans being infected with *Ophiocordyceps* sp. and turning into zombies, as imagined in the video game ‘The Last of Us’, may need reinforcing.

Visiting speakers enable interaction of children with professional microbiologists, especially in standard classes rather than large assemblies. However, many more school children can be reached if more microbiologists prepare and share educational videos. The educational benefits could be enhanced by the involvement of production companies and teachers to assist with the aesthetic and content. Such online videos hand more control to the teachers, who can embed the video material into the curriculum at the most appropriate time, pause the video and discuss key points (or deal with discipline issues). The topic of such videos could include microbiology excursions and present a phenomenon around which the rest of the presentation pivots, e.g. how to get fuel from your faeces. Engagement of commercial enterprises in the production of visual aids will require financing from grant agencies and philanthropic sources, who are encouraged to dedicate resources for such purposes.

On the other hand, interaction with scientists can be achieved remotely, which is particularly valuable for isolated communities. Notable mechanisms for doing this are ‘I’m a scientist get me out of here’ and ‘Skype a scientist’. Both mechanisms give the opportunity to ask questions, better understand what being a scientist is like and learn that scientists come from all walks of life, thereby raising aspirations of children. The latter matches scientists with classrooms across the globe and could be used to integrate the scientist as a virtual participant into parts of a school field trip.

**Fig. 18:**
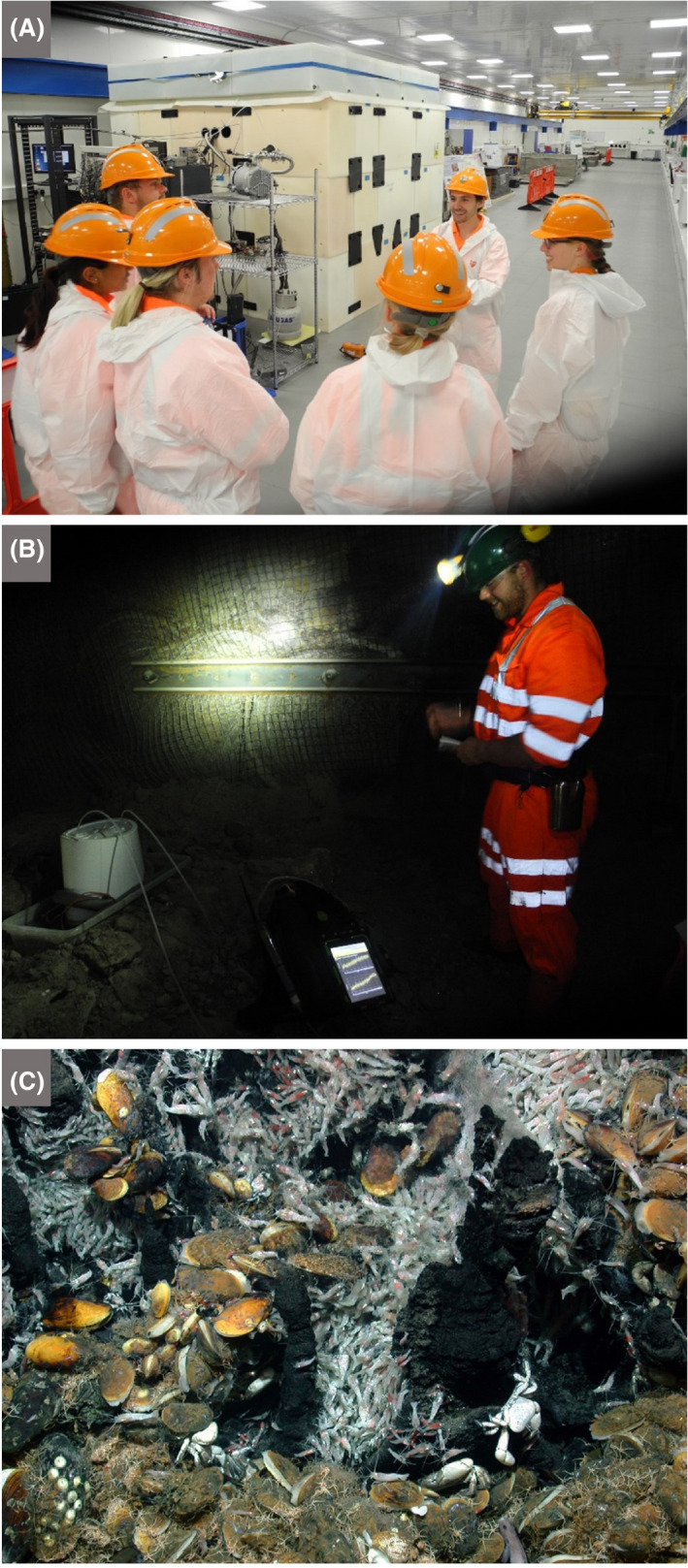
Remote field locations and research facilities can be brought to life for children, e.g. by using live feeds to schools A. Students visiting the STFC Boulby Underground Laboratory, where the focus is low background radiation science, including the search for Dark Matter. In addition, the laboratory contains clean rooms in which the concentration of airborne particles (including microbes) is minimized, together with a deep underground astrobiology facility. B. Students measured methane flux in salt‐saturated ponds constructed in the salt mine’s ‘Mars Yard’. C. Living community at hydrothermal seeps on the mid‐ocean ridge at a water depth of 3,030 metres. This diverse animal community relies on microbes, primarily Bacteria, which grow by using gases that emanate from hydrothermal vents. For example, Bacteria use sulfide (or hydrogen) to gain energy and carbon dioxide to produce biomass and organic matter that support the food web. Such microbes are often found living symbiotically in the gill chamber of the clams and shrimp. This type of symbiosis between animals and chemolithoautotrophic microbes is very common, not only in unusual environments like hydrothermal vents (where geothermally heated water and gases escape from fissures) and cold seeps (where the efflux is more diffuse and cooler than in hydrothermal vents), but also more normal environments like intertidal marine sediments (Dubilier *et al.*, 2008). New techniques provide unprecedented visual insight at the micrometre scale into such host–microbe symbioses and their metabolic interactions (Geier *et al.*, 2020). *Photographs A and B by Terry McGenity and C by MARUM – Center for Marine Environmental Sciences, University of Bremen (CC‐BY 4.0).*

Some field locations are remote or difficult to access, in which case live feeds, e.g. via Facebook, can instil some of the excitement of the microbiological activity. For example, a highlight of a recent postgraduate training school on the Deep Hypersaline Biosphere (funded by the EU COST Action MedSalt) was a descent of over a kilometre into Boulby salt mine and Boulby Underground Laboratory (Fig. 18). The visit coincided with the Mine Analogue Research (MINAR) event at which tools were being tested in the salt mine’s ‘Mars Yard’ for future planetary exploration. Subterranean interviews and demonstrations were shared using live feeds to schools and colleges across the world, illustrating children’s intrinsic interest in astrobiological research (Cockell *et al.*, 2019). Oceanographic research institutes often film and make freely available, sometimes in real time, their research trips, including deep‐sea dives by submersibles (Fig. 18).

In future, more field expeditions into unusual microbial habitats should, where possible, be financed, planned and executed in such a way that an educational component can be accomplished, and the excitement and scientific achievements of the expedition shared with children of different ages. Virtual reality and augmented reality are now feasible, extra‐sensory options for schools, as simple viewers costing $5‐10 apiece, coupled with free virtual reality apps on smartphones, allow an immersive experience of expeditions, either ready‐made (e.g. from Google Expeditions) or home‐made (Minocha *et al.* 2018).

## Discussion

### Class excursions bring microbes and their activities alive and fuel the excitement of discovery

A basic knowledge of microbiology is an essential enabler of some of the new policy decisions that will be necessary to attain sustainable development of human societies and to reverse current unsustainable practices entrained by previous inadequately informed decisions, so it is imperative that society becomes microbiology‐literate (Timmis, *et al*, 2019). For society to attain microbiology literacy, relevant aspects of microbiology must become an essential component of basic education. Happily, the breadth, depth and excitement of microbiology, along with its dynamic research advances, and especially new discoveries about our microbiomes, make it a perfect topic for stimulating curious young minds. Microbes are pervasive in their influence on us and our environment, and the results of microbial activities are highly tangible for children – witness the number of products in shops that have a microbial origin or component. Almost certainly as a consequence, there is a considerable body of experience that testifies to the fact that exposure of children to microbiology can ignite a veritable passion for the subject. The primary goal of this Editorial is to promote class excursions as a powerful means of nurturing interest in microbes, through enabling children to experience and engage with the microbial world in a very individual, personal and intimate manner. However, other, equally important goals are: to engage teachers and stimulate them to consider the value of class excursions, to recognize and encourage those academics already involved in bringing microbiology to children in diverse settings, to induce others to participate in the introduction of microbiology in basic education, and to support schools in their endeavours (Redfern *et al.*, 2013). *With this Editorial, we seek to motivate schools to expand their portfolio and frequency of excursions, and academic microbiology groups to share more ideas, resources and time to allow a wider audience – children and adults – to experience microbes and microbiologists in action.*

### Microbiology excursions cross discipline boundaries and can benefit the learning of other subjects

The examples provided in this Editorial may be centred on microbiology, but none will be exclusively so, and nor should they. Excursions afford the opportunity to explore societal, cultural and civilizational issues, both global (e.g. feedstocks used in the processes discussed, and issues surrounding their trade, transport and carbon footprint) and local (e.g. regional pride in the products, local employment opportunities and environmental impact of the processes), all of which are key components of any geography syllabus. Indeed, excursions can demonstrate how integration of knowledge and understanding from a range of other disciplines are essential for successful businesses, drawing on: engineering, physics, mathematics (e.g. design and construction of fermenters for production and recovery of microbial products such as enzymes and amino acids, calculating/controlling hydrodynamics of water flow in wastewater treatment), chemistry (e.g. identifying flavour compounds in food microbiology), biology (e.g. human health, the mode of action of drugs, how enzymes work, molecular biology tools such as gene editing and the polymerase chain reaction, created through innovative microbiology research), economics (e.g. long‐term economic viability of companies and diversification, by turning waste into profitable material), history (e.g. the role of the industry in the growth and identity of the region; how microbial infections have contributed to past events, including the eradication of civilizations), social sciences and psychology (e.g. the nature and role of stakeholders in all sorts of issues, such as the use of growth promoters in food animal husbandry and impacts on drug resistance in pathogens, human behaviours in response to pandemics, societal and logistical challenges associated with testing for pathogens), technology (e.g. how key infrastructure, such as clean rooms and fermentation tanks, works and is controlled), art and design (e.g. how a product is marketed, degradation and restoration of art objects from ancient manuscripts to cinematographic film), and ethical issues (e.g. industrial carbon footprint, animal testing and welfare). Excursions should thus imbue children with a sense that the most rewarding jobs will require them to be adaptable and integrate ideas from a range of subjects.

The corollary of this is that microbiological understanding collaterally supports understanding in a range of other disciplines. Consequently, excursions that are not primarily designed to have a microbiology focus can still present the excitement of the invisible world of microbes to children, as exemplified in a number of the discussion topics associated with the suggested excursions.

Most importantly, and especially because microbiology excursions can incorporate multiple topics, they can expose/elevate awareness of local issues and global Grand Challenges, provide unique opportunities to explore and discuss them, instil a sense of community‐global responsibility and encourage participation in collective efforts that seek solutions.

### Microbiology excursions can facilitate microbial awareness and improve learning by providing a multisensory experience

It is well known that stimulating multiple senses enhances learning (Shams and Seitz, 2008), and excursions provide ideal situations in which microbes and their activities can be seen, touched, smelled, sometimes tasted, and occasionally heard. Visual manifestations of microbial agglomerations and their activities are of central importance in learning about microbiology, and many examples are scattered throughout this Editorial. But, there are more ways by which microbes and their activities can stimulate our senses, as outlined here.

#### Sight

**Fig. 19:**
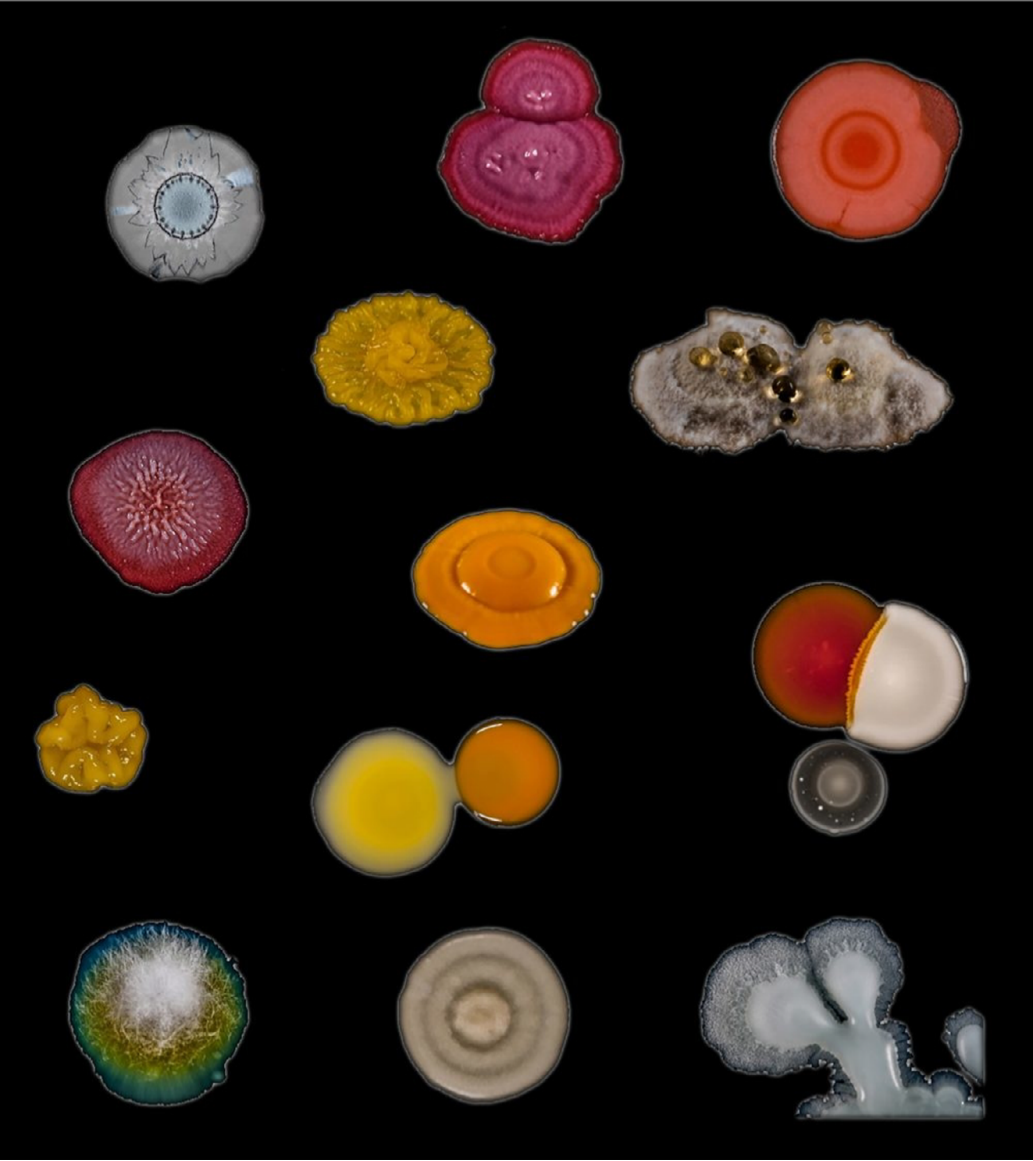
An array of microbial colonies growing on agar plates, demonstrating different forms, textures and colours. Beautiful images of microbes and their habitats, along with interesting context, can be found in the book called ‘Life at the Edge of Sight’ (Chimileski and Kolter, 2017). *Photograph by Scott Chimileski and Roberto Kolter*

The microbes within a school pond, for example, will only really come alive for children when they can see them at the single‐cell level under a microscope or stereomicroscope, i.e. when they can discover microbes’ relative sizes, cell division, motility and how they are eaten by protozoa and small invertebrates. Children usually love visual action – the more frenetic, the better – and there are few examples in nature with more frenetic action than the microbial goings‐on in pond water. Microscopic examination of glass slides left in a pond over time will enable children to observe microbial colonization and biofilm production, and thus explore ecological concepts such as succession, which is normally exemplified by larger organisms. It is also enjoyable and educational for children to observe microbial colonies of different forms, textures and colours growing on agar plates inoculated with samples they have taken during an excursion (Fig. 19).

#### Touch

Touching a slimy pebble from a pond or stream should initiate discussions about biofilms, including which microorganisms create them, what is the chemical nature of the slime, what benefits the slime provides for the microbes producing it and perhaps other microbes embedded in it. Any disgust exhibited by some children may be tempered (or perhaps enhanced) by pointing out that they will certainly have eaten microbial slime with considerable pleasure. For example, the texture of many varieties of ice cream is enhanced by xanthan gum, a high molecular weight polysaccharide produced by the bacterium *Xanthomonas campestris*. Children may then consider where else they find biofilms: trickling filters in wastewater treatment plants, air pollutant biofilters, snottites in caves (Macaldy *et al.*, 2007), the bottoms of shower curtains, taps and unwashed sinks, the surfaces of drainpipes, teeth and catheters. The discussion could be extended to consider the ubiquity of biofilms, their benefits to the environment and health (e.g. the ‘second skins’ of plants and animals that protect against pathogens, biofilms in waste and pollution treatment) along with their harmful effects (e.g. steel corrosion, tooth decay, as well as housing pathogens and protecting them against antimicrobials and disinfectants).

#### Smell and taste

Children have an acute awareness of smell, so exploration of microbial odours is a powerful way to relate microbes to sensory perception. In some cases, it could be interesting to ask children ‘what are smells and how do we detect them?’. Odours are volatile chemicals that are recognized by olfactory receptors in the nose (it has been suggested that we can distinguish up to one trillion different odours; Bushdid *et al.*, 2014). Volatiles are not only important for us to anticipate and appreciate a nice curry or react to unpleasant or dangerous situations, such as food that has gone bad or a gas leak, but are used by microbes to communicate among themselves and with other organisms, in order to orchestrate some joint action (Ryu *et al.*, 2003). To get children involved, it can be helpful to pose the question: ‘Can I smell microbes?’ and then to ask them to identify smells they are familiar with, perhaps categorized into nice and unpleasant odours, and then discuss which of these has a microbial origin. Body odours (also from pets) emanating from poo, flatulence, old sweat, smelly (‘cheesy’) feet (see comments on *Propionibacter* and *Brevibacterium*, below) and bad breath are all produced by microbes when they metabolize chemicals in the particular body sites they inhabit. Volatile chemicals produced by microbes from secreted oils in the axillae have been considered to have pheromone properties, and so the microbiome may act like an invisible puppet master in attracting mates. Volatiles in breath are currently being actively investigated as biomarkers of disease for non‐invasive diagnostic procedures.

Outside, we have the nice smells of rotting leaves on the ground in autumn and compost heaps all the year‐round and, of course, the smell of soil after rain, caused by geosmin‐producing *Streptomyces* species, to which we alluded above. The bad‐egg smell derived from stirring up the black mud at the bottom of a pond will likely evoke a memorable response from children. Here, it is informative to discover which microbes produce the smell (sulfate‐reducing bacteria), what is the chemical involved, how it may be created (for older children having done some chemistry), where the responsible microbes live and why they may be beneficial or harmful in different scenarios. This activity may be coupled with digging a small pit, in which the redox zonation in sediments may be seen in the form of microbially induced changes in the colour of iron minerals, from aerobic (rusty) to sulfate‐reducing/sulfidogenic (grey‐black) (Fig. 13). The evocative smell of the sea can serve as the basis for a discussion on biogenic volatile chemicals, primarily dimethylsulfide (DMS), which is produced by microbial breakdown of dimethylsulfoniopropionate, a multifunctional metabolite found in many marine microbes that can serve as an osmolyte, antioxidant and cryoprotectant. Discussions could then turn to the role of DMS in cloud condensation and potentially global cooling, and even how seabirds use DMS as a directional olfactory cue (Steinke *et al.*, 2011).

In the home, there are well‐known odours produced by microbes, including those coming from food, like cheese (see below), sauces obtained by fermentation, vinegar and pickles of all sorts, and fermented meats. The same smells can be experienced in restaurants and food stores. Shops and stalls selling fish may have the typical odour of fish that is not super fresh. This is trimethylamine, a pungent chemical produced by microbes living on/in the fish from trimethylamine oxide, a versatile chemical made by fish to protect against the protein‐destabilizing effect of urea, salinity and high pressure (Yancey, 2005). The flavours of food definitely provide an enjoyable way for children to learn about microbes. Cheeses, for example, derive from coagulated milk, yet they can taste and smell differently and have a range of textures and skins, reflecting the different microbes and processes used in their manufacture. The microbial communities that develop are a consequence of, *inter alia,* the microbial inoculum, the cheese ‘environment’, e.g. humidity, salinity, temperature and pH. The fungus, *Penicillium roqueforti*, grows in the cracks made in cheeses like Stilton (Fig. 3) and Roquefort, giving the distinctive earthy flavour and blue veins (Kindstedt, 2014). Microbial fermentation products give interesting flavours and textures, e.g. the bacterium *Propionobacter* sp. produces CO_2_ (the holes) and acetate/propionate in typical Swiss alpine cheeses – the same microbial genus also contributes to the unwashed smell in humans. Similarly, *Brevibacterium linens* that grows on the surface of some cheeses also grows under our toenails, producing a distinctive smell common to both. Thus, from cheese, the discussion can turn to the microbial origin of the human aroma and its influence in attracting a mate.

#### Sound (and vision)

Sound is not a sense we commonly associate with microbes. However, the Fermentophone (Fig. 16) harnesses bubbles from colourful fermentations to create music. Bubbles can also be seen and sometimes heard to pop when escaping from the water column to the atmosphere. Children will often ask what causes the bubbles and the answer will depend on various factors including illumination. But, in a stagnant muddy pond it is likely that bubbles from the sediment are formed from methane, which can stimulate discussions about methane’s role as a greenhouse gas. Showing children videos of burning methane emanating from a pond is highly recommended, with the common outcome of conversations turning to personal methane production and its egress, thus providing a link between the individual and their microbiome, specifically in this case their archaeome (Fig. 13).

#### Indirectly sensing microbial activity

Measuring the temperature of a school compost pile over time provides another means of sensing microbial activity that can feed discussions about energetics, decay and microbial interactions with other species, such as compost worms, which may be considered to be environmental engineers, and their all‐important gut microbes which carry out many of the compost digestive processes (Medina‐Sauza *et al.*, 2019). Children could use a thermometer to accurately record changes in temperature or simply use touch to sense microbial activity by feeling the warmth of a metal rod inserted into the compost heap. There are other tools to detect microbial activity; for example the *MudWatt* (Magical Microbes; Jude and Jude, 2015) provides a cheap yet very effective device to observe metal‐reducing microbes in sediments/soils making electricity and generating light.

### Microbiology excursions can be supplemented with a range of other activities to reinforce learning

**Fig. 20:**
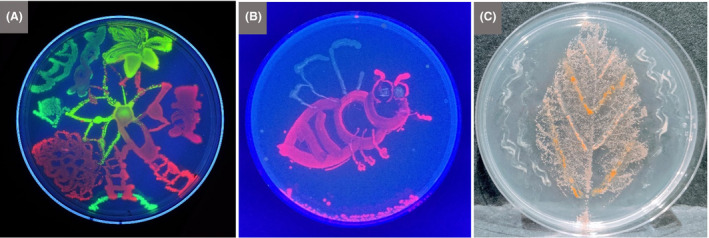
Agar Art. The top three entries in the American Society for Microbiology (ASM) Agar Art Kids 2019 competition A. ‘Circle of Life’ by Kate Lin (Age 11) using *E. coli* MM294 pGFPuv (green), pYellow (yellow), pCherri (purple) and pTang (Pink) growing on LB Agar + Ampicillin, with support from Cold Spring Harbor Laboratory DNA Learning Center. B. ‘The Honey Bee’ by Manal Faisal Khan (Age 5) using *E. coli* growing on Nutrient Agar. C. ‘Fall’ by Lilu Good‐Martinez (Age 10) using *Methylobacterium extorquens* growing on minimal medium with methanol as a carbon source. Congratulations to the entrants named above and thanks to them and the ASM (https://asm.org/Press-Releases/2019/November-1/ASM-s-5th-Agar-Art-Contest-Showcases-the-Beauty-of) for these images. The ‘Microbial Art’ website and the June 2017 edition of SfAM’s magazine, *Microbiologist*, provide many examples of the interplay between art/fashion and microbiology, and Park (2012) provides practical tips for creating microbial art.

**Fig. 21:**
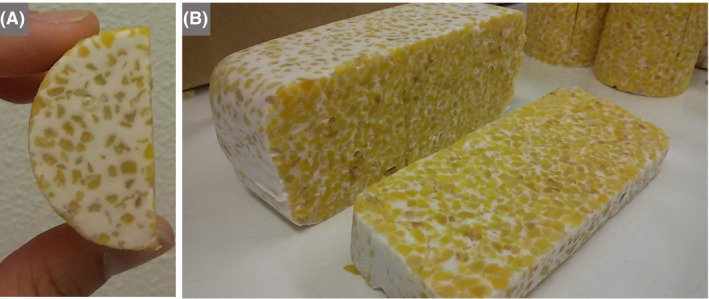
Classroom activity making lupin‐based tempeh. Tempeh is a fermented Indonesian food, usually made from soya beans. Here, the soya beans have been replaced with lupin beans, e.g. from *Lupinus angustifolius*. The fungus, *Rhizopus oryzae*, makes a white mycelial network knitting together the lupin grits into sliceable tempeh, ready for marinating or frying. These slices contain vitamin B_12_ thanks to the use of a co‐culture of the *Rhizopus oryzae* with the vitamin B_12_‐producing food‐grade bacterium *Propionibacterium freudenreichii*, thus making this specific type of lupin tempeh an excellent replacement of meat (Wolkers‐Rooijackers *et al.*, 2018). Children can inspect the spores of *Rhizopus oryzae* before using them to inoculate the soaked lupin grits. It takes 3 days of incubation before the ‘tempeh burgers’ are ready for cooking. *Photograph by Martha Endika*

For all excursions, learning can be reinforced in the classroom with follow‐up activities: designing posters, presenting news reports, building models and other artwork and including agar art, which involves developing images using microbes inoculated onto agar plates (Charkoudian *et al.*, 2010; Adkins *et al.*, 2018; Fig. 20). The significance of a visit to a bakery, for example, would be enhanced by investigating gas production (using balloons) and pH change (using indicators) in yeast cultures back in the classroom. After exploring microbial contributions to foods in shops, manufacturing facilities or research laboratories, it is fun and instructive for children to make their own fermented foods, such as tempeh (Fig. 21). Samples transported back to the class can become the subject of observation/investigation/experimentation. These activities are facilitated by tools like the *Foldscope* (Cybulski *et al.*, 2014), together with a range of open‐source technology and products, such as centrifuges (Bhamla *et al.*, 2017) and nephelometers (Wijnen *et al.*, 2014). Samples of sea salt, salted fish or salt‐rich fermented food can be enriched in medium to grow extreme halophiles, such as *Halobacterium salinarum*, which is readily cultivated, safe to work with and is a striking red colour (Baxter *et al.*, 2012; DasSarma *et al.*, 2016; Fig. 2). Winogradsky columns provide another relatively simple means of exploring the different ways in which microbes obtain energy, their interactions, succession and niche partitioning, using mud and water from local environments (Anderson and Hairston, 1999; Fig. 13). The Microbiology in Schools Advisory Committee (MISAC), which has been established for 50 years, provides outstanding and wide‐ranging educational resources, many of which have been adopted worldwide, for those keen to teach microbiology in schools. Obviously, any classwork involving the cultivation of microbes should be carried out according to Good Microbiological Laboratory Practice (see the online guide by the Microbiology Society).

### Summary and recommendations

**Fig. 22:**
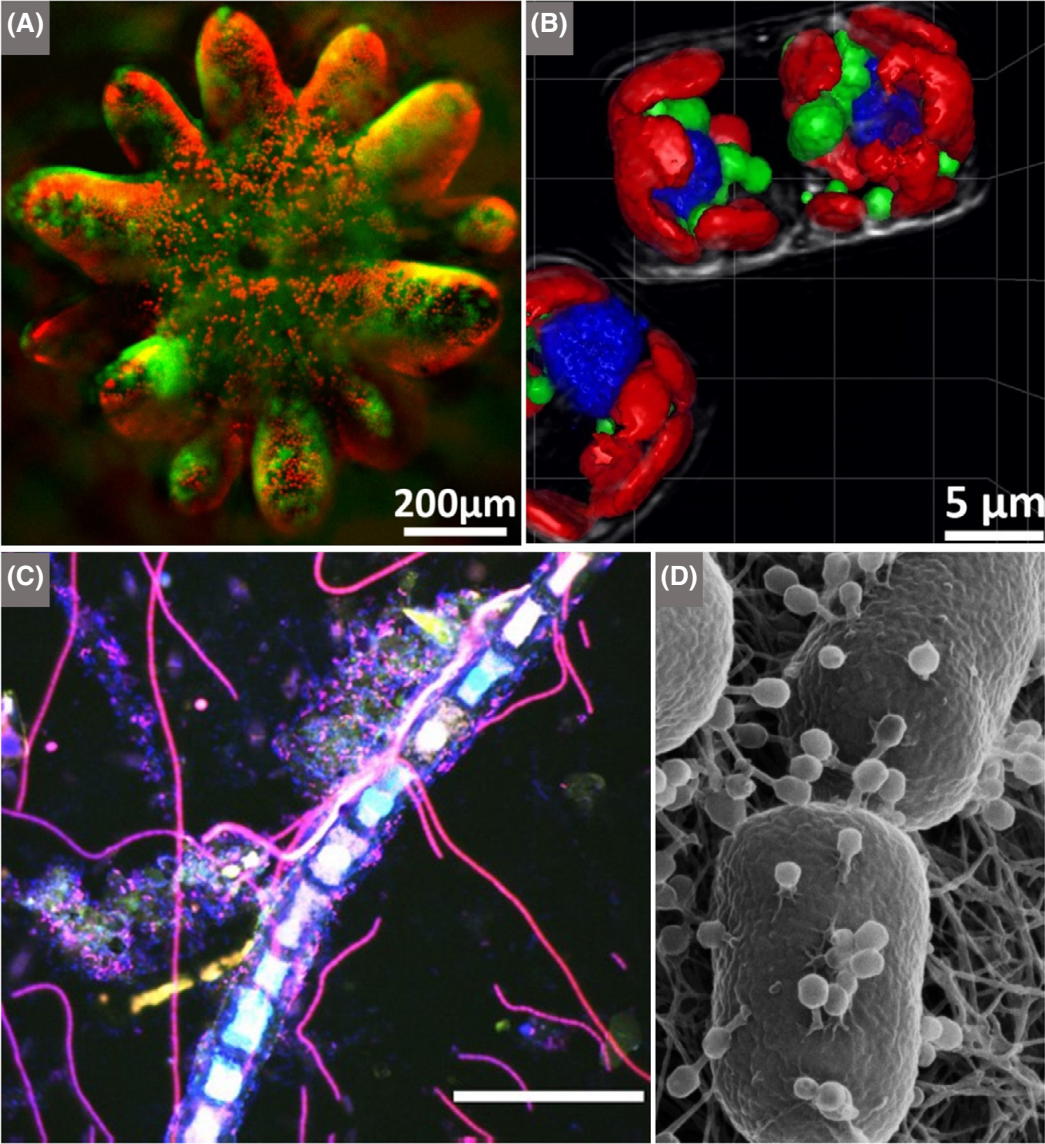
New and diverse imaging techniques make microbes more attractive to broadcasters and publishers A. Fluorescence micrograph of a coral polyp, showing autofluorescence of the coral tissue (green) and the photosynthetic *Symbiodinium* algae living inside (red). To image these photosensitive corals, a custom light‐sheet microscope was made. This image won the 2019 Nikon Small World in Motion Competition, and method details can be found in Laissue *et al. *(2019). B. Fluorescence micrograph of the diatom *Thalassiosira nanae*. Chloroplasts are red, neutral lipids green, DNA blue and the cell wall white. Fluorescence microscopy provides three‐dimensional information of individual components, enabling the measurement of their volumes. For more information, see Chansawang *et al. *(2016). C. Fluorescence in situ hybridization image of coral reef biofilms using confocal microscopy. Samples were hybridized with the Cy5‐labelled Bacteria‐specific probe (EUB338), the Cy3‐labelled GAM42a (for Gammaproteobacteria), the fluorescein‐labelled ALF1b (for Alphaproteobacteria) and the Cy3‐labelled Arch915 (for Archaea). Cells which appear magenta are Gammaproteobacteria*,* cyan cells are Alphaproteobacteria*,* blue cells are other Bacteria, and red cells are Archaea. Large autofluorescent algal filaments can also be observed interspersed with the smaller bacterial and archaeal cells. The scale bar is 100 µm. D. Helium ion microscopy image showing T4 phage infecting *E. coli.* Some of the attached phage have contracted tails indicating that they have injected their DNA into the host. The bacterial cells are ~ 0.5 µm wide, equivalent in size to the small dots seen in C. For more information, see Leppänen *et al. *(2017). Time‐lapse imaging and videos of live organisms (e.g. A), rotatable images (e.g. C), and realistic graphics coupled with high‐resolution imaging to demonstrate relative position and very fine detail from nanometre to micrometre scales (brilliantly exemplified by using cryo‐electron microscopy to illustrate cell surface structures in *Caulobacter crescentus* by von Kügelgen *et al. *(2019)), all add an extra dimension that can help to attract children to the wonder of microbes, their structures, activities and interactions. Goodsell *et al. *(2020) review the plethora of techniques that are allowing cellular machines, compartments and macromolecules to be visualized, and they consider the important issue of the use of artistic licence, particularly to stimulate interest and to educate the non‐technical audience. *Photographs A and B by Philippe Laissue, C by Nicole Webster, D by Miika Leppänen (permission from Wiley).*

There is an urgent need for society to acquire literacy in microbiology, and the education and inspiration of children lie at the heart of this endeavour. Pivotal to this are actions that will enable children to ‘see’ invisible microbes, by experiencing their activities and consequences of such activities. Here, we have discussed the importance of class excursions to experience and explore microbes in action, provided a range of examples, and have suggested some organizational issues that may be helpful for obtaining maximum benefit from such excursions. To advance the goal of initiating class excursions in microbiology, we make the following recommendations:

- Schools to implement an explicit policy of class excursions, during which microbial activities can be experienced and relevant topics explored
- School Heads to incentivize teachers to arrange excursions and interactions with scientists, especially with a view to raising students’ aspirations
- Policymakers to support schools in developing exciting and educationally valuable excursions, by providing flexibility in the curriculum and the modest enabling financial resources
- Government Education Ministries to ensure all schools are equipped with microscopes
- Commercial organizations, or clusters of organizations, government agencies and departments, and NGOs that require a microbiology‐literate workforce, to contribute financially, materially and politically to support microbiology education in schools, e.g. following the model of https://novonordiskfonden.dk/en/projects-and-initiatives/life/
- Universities, agencies and organizations to introduce/implement explicit support and reward for employees (e.g. by including outreach as a promotion criterion) who embark on activities that improve science/microbiology literacy, especially with a view to widening participation
- Microbiology practitioners, academics and teachers to coordinate and enhance dialogue, leading to the development of free online educational materials that best fit with student and teacher needs (e.g. via microbiology society conferences)
- Universities and research institutions to be encouraged to set up microbiology‐related (mobile) exhibitions/activities or themed museums for education
- Funding bodies (research councils, learned societies, etc.) to provide adequate grant funding to support innovative applications from microbiologists to get children involved in immersive, active learning outside of the classroom
- Annual meetings of learned societies to include an open‐door session aimed at schools and the general public, with hands‐on activities
- Microbiology societies to include the promotion and facilitation of class excursions in the remit of their outreach ambassadors
- Museums (as well as zoos, aquariums and botanical gardens) to grow their remit of promoting and contributing to Sustainable Development Goals by increasing the visibility of microbes and their ecosystem functions/services
- Broadcasters to accelerate the inclusion of microbes into wildlife documentaries commensurate with their importance to the planet, taking on board the numerous ways by which they act on the senses described above, together with exceptional advances in imaging (Fig. 22).

## Conflict of interest

None declared.

**Links to the resources mentioned or alluded to in the text**

- Albuquerque Bernalillo County Water Utility Authority: Water Resources Education http://www.abcwua.org/education
- Alexander Fleming’s Museum in London https://www.imperial.nhs.uk/about-us/who-we-are/fleming-museum
- American Museum of Natural History: Bacteria in the Cafeteria game https://www.amnh.org/explore/ology/microbiology/bacteria-in-the-cafeteria-game
- Bacterial World exhibition at the Oxford Museum of Natural History https://microbiologysociety.org/blog/bacterial-world-exhibition-at-the-oxford-museum-of-natural-history.html
- Beijerinck Museum in Delft https://www.delta.tudelft.nl/article/whats-hiding-beijerinck-museum
- Big Compost Experiment https://www.bigcompostexperiment.org.uk
- Bioluminescent Photograph Booth http://www.annebrodie.com/exploring-the-invisible-new/2014/9/22/bioluminescent-photograph-booth
- Centre for Disease Control: Solve the Outbreak game https://www.cdc.gov/mobile/applications/sto/web-app.html
- Comic Bacteria’s Micro‐Match game https://www.comicbacterias.com/micromatch
- The Council for Learning Outside the Classroom (LOtC) https://www.lotc.org.uk
- DTU Bioengineering’s Biotech Academy http://www.biotechacademy.dk
- The Eden Project – Invisible Worlds https://www.edenproject.com/learn/for-everyone/invisible-worlds-massive-open-online-course-mooc
- Exploring the Invisible https://exploringtheinvisible.com
- Foldscope https://www.foldscope.com, with a background video https://youtu.be/ky-cqSI5mwE and information about how to share microscopic discoveries microcosmos.foldscope.com
- Foldit https://fold.it/portal/
- Good Microbiology Laboratory Practice guide from the Microbiology Society https://microbiologyonline.org/teachers/safety-information/good-microbiological-laboratory-practice
- I’m a scientist get me out of here https://imascientist.org.uk
- Instituto Butantan Museum of Microbiology in Brazil http://www.butantan.gov.br/atracoes/museu-de-microbiologia
- International Genetically Engineered Machine (iGEM) Competition https://igem.org/Main_Page
- International Microorganism Day https://fems-microbiology.org/international-microorganism-day/
- International Ocean Discovery Program (IODP) educational resources http://iodp.tamu.edu/outreach/education.html
- Justine Dees’ Joyful Microbe blog: https://justinedees.com/blog
- Kew Gardens – Protecting precious fungi https://www.kew.org/read-and-watch/protecting-precious-fungi
- Louis Pasteur’s house in Arbois http://www.terredelouispasteur.fr/en/louis-pasteurs-house
- La Main à la Pâte resources on inquiry‐based learning in science and technology: https://www.fondation-lamap.org/en/international
- Microbial Art, a collection of art by scientists and artists from around the world http://www.microbialart.com
- Microbial Evolution and Growth Arena (MEGA) plate https://www.youtube.com/watch?v=6mUqbToqAM0
- Microbiology in Schools Advisory Committee (MISAC) http://misac.org.uk/anniversary-articles.html
- Microbiota exhibition at La Cité des Sciences in Paris http://www.cite-sciences.fr/en/ressources/expositions-passees/microbiota/the-exhibition
- Micropia Museum in Amsterdam https://www.micropia.nl/en/visit/what-is-micropia/museum-microbes
- Museum at the Robert Koch Institute in Berlin https://www.rki.de/EN/Content/Institute/Museum_Mausoleum/museum_en.html
- Mushrooms primary school activity pack – BBSRC https://bbsrc.ukri.org/documents/mushroom-pdf/
- The Novo Nordisk Foundation, LIFE https://novonordiskfonden.dk/en/projects-and-initiatives/life
- One Health https://www.who.int/features/qa/one-health/en
- Outbreak exhibition at the Smithsonian Museum of Science https://naturalhistory.si.edu/exhibits/outbreak-epidemics-connected-world
- Public Health England’s e‐Bug initiative https://www.e-bug.eu
- Skype a scientist https://www.skypeascientist.com/talk-to-a-scientist.html
- Small World Initiative http://www.smallworldinitiative.org
- SySTEM 2020, a European network that focuses on science learning outside the classroom: https://www.ecsite.eu/activities-and-services/projects/system-2020
- Tiny Earth https://tinyearth.wisc.edu/
- This is Microgeography: Exploring the overlooked but ubiquitous microbiology of our urban landscapes https://microgeography.wordpress.com
- UK Fungus Day https://www.ukfungusday.co.uk/join/schools
- UN Sustainable Development Goals https://sustainabledevelopment.un.org
- Wellcome Trust Collection in London https://wellcomecollection.org
- *Wolbachia* project https://www.vanderbilt.edu/wolbachiaproject
- World in a drop: photographic explorations of microbial life in Harvard Museum of Natural History https://hmnh.harvard.edu/world-drop-photographic-explorations-microbial-life
